# Multiomic analysis of cohesin reveals that ZBTB transcription factors contribute to chromatin interactions

**DOI:** 10.1093/nar/gkad401

**Published:** 2023-06-02

**Authors:** Rui Wang, Qiqin Xu, Chenlu Wang, Kai Tian, Hui Wang, Xiong Ji

**Affiliations:** Key Laboratory of Cell Proliferation and Differentiation of the Ministry of Education, School of Life Sciences, Peking-Tsinghua Center for Life Sciences, Peking University, Beijing 100871, China; Key Laboratory of Cell Proliferation and Differentiation of the Ministry of Education, School of Life Sciences, Peking-Tsinghua Center for Life Sciences, Peking University, Beijing 100871, China; Key Laboratory of Cell Proliferation and Differentiation of the Ministry of Education, School of Life Sciences, Peking-Tsinghua Center for Life Sciences, Peking University, Beijing 100871, China; Key Laboratory of Cell Proliferation and Differentiation of the Ministry of Education, School of Life Sciences, Peking-Tsinghua Center for Life Sciences, Peking University, Beijing 100871, China; Key Laboratory of Cell Proliferation and Differentiation of the Ministry of Education, School of Life Sciences, Peking-Tsinghua Center for Life Sciences, Peking University, Beijing 100871, China; Key Laboratory of Cell Proliferation and Differentiation of the Ministry of Education, School of Life Sciences, Peking-Tsinghua Center for Life Sciences, Peking University, Beijing 100871, China

## Abstract

One bottleneck in understanding the principles of 3D chromatin structures is caused by the paucity of known regulators. Cohesin is essential for 3D chromatin organization, and its interacting partners are candidate regulators. Here, we performed proteomic profiling of the cohesin in chromatin and identified transcription factors, RNA-binding proteins and chromatin regulators associated with cohesin. Acute protein degradation followed by time-series genomic binding quantitation and BAT Hi-C analysis were conducted, and the results showed that the transcription factor ZBTB21 contributes to cohesin chromatin binding, 3D chromatin interactions and transcriptional repression. Strikingly, multiomic analyses revealed that the other four ZBTB factors interacted with cohesin, and double degradation of ZBTB21 and ZBTB7B led to a further decrease in cohesin chromatin occupancy. We propose that multiple ZBTB transcription factors orchestrate the chromatin binding of cohesin to regulate chromatin interactions, and we provide a catalog of many additional proteins associated with cohesin that warrant further investigation.

## Graphical Abstract

**Graphical Abstract:**
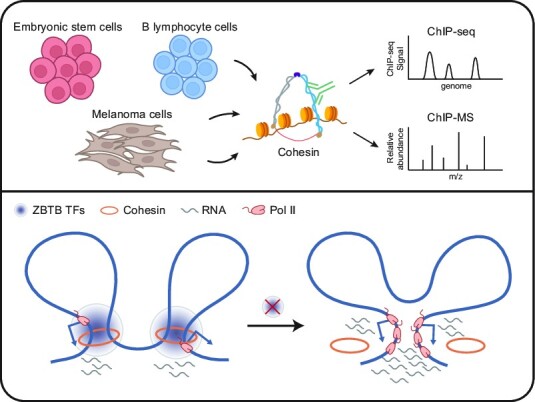

## INTRODUCTION

Chromatin structures are partitioned into hierarchical units, including chromosome territories, A/B compartments, topologically associating domains (TADs), enhancer–promoter loops, nucleosome clutches, globular chromatin domains and nucleosomes (1–12). Although enhancer- and promoter-mediated local chromatin architectures have been studied for several decades (13–18), the molecular basis of their establishment, maintenance and disruption remains unclear. To describe these mechanisms, the looping model, tracking model and phase separation-based compartmentalization model have been introduced (19–22). Many different chromatin structural regulators have been depleted in different studies, resulting in different effects on 3D genome organization (23–27). The dissection of these molecular mechanisms is crucial to understanding the regulation of the 3D genome during development and disease.

Structural maintenance of the chromosome (SMC) complexes (cohesin, condensin and SMC5/SMC6) are crucial for the organization of interphase chromatin structures (28–30). Previous genomic distribution mapping efforts suggested that cohesin occupies promoters, enhancers and insulators (31–33). Cohesin is loaded by NIPBL and MAU2, stabilized by CTCF at insulators, and removed by WAPL (34–36). These direct regulators of cohesin have been shown to regulate global 3D genome organization (37–40). Previous studies have identified multiple transcription factors involved in 3D genome organization that interact directly or indirectly with cohesin. These factors, including CTCF, YY1, ZNF143, ADNP, MAZ, WIZ and BHLHE40 (41–51), usually occupy insulators or gene promoters, and their perturbation modulates the global 3D chromatin organization or affects a subset of chromatin interactions. We hypothesized that there are additional transcription factors involved in cohesin-mediated chromatin interactions. Therefore, the comprehensive identification of cohesin-interacting partners will provide a valuable resource for the identification of candidate regulators of 3D genome organization and will support the idea that the redundancy between cohesin and various transcription factors modulates local chromatin interactions or specific interactions under different conditions.

The ZBTB transcription factors Mod (mdg4) and Cp190 are known to play critical roles in the regulation of insulator activities in *Drosophila* (52–56). The zinc finger domain interacts with specific DNA sequences, and the BTB domain mediates homo- and heteromeric interactions (57,58). These properties might be responsible for the ability of ZBTB transcription factors to mediate 3D chromatin structures. There are >40 ZBTB transcription factors in mammals, and many of them have been reported to function in T-cell and B-cell lineage specification during development and diseases (59–62). The major focus was previously on their roles in transcription regulation. However, whether and how they regulate 3D chromatin structures remain unknown.

Here, we report that ZBTB21 facilitates the chromatin occupancy of cohesin and modulates 3D chromatin interactions and transcriptional repression. A number of ZBTB transcription factors (ZBTB7A, ZBTB7B, ZBTB11 and ZBTB35) have been shown to interact with cohesin. The double depletion of ZBTB21 and ZBTB7B further decreases the chromatin occupancy of cohesin. These results imply that various ZBTB transcription factors facilitate cohesin chromatin binding in addition to performing transcriptional regulatory activities that modulate chromatin interactions in mammalian cells.

## MATERIALS AND METHODS

### Cell culture

Mouse embryonic stem cells (mESCs) (obtained from Richard Young's lab, MIT) were cultured in knockout Dulbecco’s modified Eagle’s medium (DMEM; Gibco; 10829018-018), supplemented with 15% fetal bovine serum (FBS), PenStrep (Gibco, 15140-122), nucleoside (Sigma, ES-008), l-glutamine (Gibco, 25030-149), NEAAs (non-essential amino acids; Milipore, TMS-001-C), CHIR99021 (Selleck, S1036), PD0325901 (Selleck, S1263), β-mercaptoethanol (Sigma, M3148) and mLIF (mouse leukemia inhibitory factor; Milipore, ESG1107) at 37°C with 5% CO_2_. To degrade the target proteins, we first treated the degron-tagged mESCs with 1 μg/ml doxycycline (Sigma Aldrich, D9891) for 24 h, then added 500 μM indole acetic acid (IAA; Sigma Aldrich, 115148) for the indicated times for different analyses. HEK293T (ATCC, CRL-3216) cells were cultured in DMEM (Gibco, 11995-065) supplemented with 10% FBS (Gibco, 10099-141) and PenStrep (Gibco, 15140-122) at 37°C with 5% CO_2_.

### Plasmid construction

The donors and single guide RNAs (sgRNAs) for the degron-tagged cells were generated as in the previous report (63). The homology arms were amplified from mouse genomic DNA with a length of 400 bp upstream or downstream of the targeted site. The homology arms were then ligated with mouse auxin-inducible degron (mAID)–green fluorescent protein (GFP) donor cassettes by Gibson assembly (NEB, E2611S). The ligated constructs were used to target the sequence near the TAG stop codon of the protein of interest by CRISPR/Cas9 gene editing to generate degron cell lines. The plasmid pMK243 (Addgene, 72835) was digested by restriction enzymes BglII and MluI (NEB, R0144S and R0198S). The coding sequences of the target protein and the GFP open reading frame (ORF) were ligated with digested pMK243 by Gibson assembly. The correct construct was used to generate Tet-on inducible expression of ZBTB stable HEK293T cell lines. For primers, see Supplementary Table S9.

### Stable cell line generation

The degron donors and corresponding sgRNAs were transfected into the mES-OsTIR1 cell line generated in a previous study (64). The transfected cells were selected with neomycin (Gibco, 21810-031), and homogenous clones were obtained after the validation by genotyping and Sanger sequencing. The HEK293T cell lines expressing a doxycycline-inducible GFP-tagged ZBTB proteins were generated by inserting the expression ORF into the AAVS1 locus by CRISPR/Cas9 gene editing. The clones were validated by genotyping and Sanger sequencing. In order to not express the ZBTB protein to very high levels, ZBTB–GFP heterozygous clones were selected for downstream analyses.

### Chromatin immunoprecipitation (ChIP)

The ChIP experiment was performed based on a previous publication (65). Briefly, the 1% formaldehyde-fixed cells were lysed by Nonidet P-40 (NP-40) lysis buffer (10 mM Tris–HCl pH 7.5, 150 mM NaCl, 0.05% NP-40). The cell lysates were added on top of a 1.25 ml sucrose cushion [24% sucrose (w/v) in NP-40 lysis buffer]. The nuclei were collected by centrifugation. The nuclear pellet was gently resuspended with 0.5 ml of glycerol buffer [20 mM Tris–HCl pH 8.0, 75 mM NaCl, 0.5 mM EDTA, 0.85 mM dithiothreitol (DTT), 50% (v/v) glycerol] and 0.5 ml of nuclei lysis buffer (10 mM HEPES pH 7.6, 1 mM DTT, 7.5 mM MgCl_2_, 0.2 mM EDTA, 0.3 M NaCl, 1 M urea, 1% NP-40) to isolate chromatin. The chromatin pellets were resuspended in 1 ml of sonication buffer [20 mM Tris–HCl pH 8.0, 150 mM NaCl, 2 mM EDTA pH 8.0, 0.1% sodium dodecylsulfate (SDS), 1% Triton X-100]. Micrococcal nuclease (MNase; 40 U) was added to the chromatin extracts to digest chromatin. The chromatin was further fragmented by sonication with biorupter with the setting at the position high 30 s on/60 s off 15 times. The 5% lysate was saved as input: half for the input of protein analysis and half for the input of DNA analysis. Different antibodies were added to the soluble chromatin extracts and incubated at 4°C with rotation overnight. The next day, Protein G Dynabeads (Thermo Fisher,10004D, #11205D) were added to the chromatin–antibody reaction and incubated for ∼2 h at 4°C. Protein G beads were washed with sonification buffer, high-salt buffer, LiCl buffer and TE buffer, respectively. The ChIP products were eluted from beads by heating at 65°C for 15 min. De-cross-linked DNA was used for ChIP-seq library preparation, and eluted protein products were used for mass spectrometry (MS) analysis. High-salt buffer (20 mM Tris–HCl pH 8.0, 500 mM NaCl, 2 mM EDTA pH 8.0, 0.1% SDS, 1% Triton X-100), LiCl buffer (10 mM Tris–HCl pH 8.0, 250 mM LiCl, 1 mM EDTA, 1% NP-40) and TE buffer (1 mM EDTA, 10 mM Tris–HCl pH 8.0).

### BAT-Hi-C

The BAT Hi-C was performed by following the protocol published previously (66). The major difference between BAT Hi-C and *in situ* Hi-C is that BAT Hi-C employs a biotin-labeled bridge linker to mediate proximity ligation, while *in situ* Hi-C uses blunt end ligation. Briefly, the 1% formaldehyde-fixed cells were digested by AluI (NEB, #R0137S) for at least 12 h. Then, the restriction enzyme was inactivated by heat. The DNA overhangs were added with a base dA by Klenow (3′–5′ exo-) enzyme [40 μl of NEB buffer 2, 8 μl of 10 mM dATP, 40 μl of 10% Triton X-100, 304 μl of H_2_O and 8 μl of Klenow (3′–5′ exo-)]. The dA overhang DNA products were ligated with a bridge linker by T4 DNA ligase (NEB, #M0202L) at room temperature for at least 6 h. Then the chromatin fractions were isolated, and the unligated DNA was digested by lambda exonuclease. The ligated DNA was de-cross-linked at 65°C overnight. The ligated DNAs were purified, and then fragmented by sonication. The fragmented DNA was examined by 1% DNA agarose gel electrophoresis. The size of the DNA product was ∼0.2–2 kb. The ligated DNA was purified by biotin–streptavidin pulldown. The DNA library was constructed with the NEBNext®Ultra™II DNA Library Prep Kit for Illumina®. The 300–500 bp DNA products were purified by the Magen gel purification kit. The DNA was sequenced at Hiseq 4000, and 150 bp paired-end reads were obtained for downstream analyses.

### 4C-seq

The 4C-seq was described in a previous publication (67). Briefly, the cells were first treated appropriately and then cross-linked with 1% formaldehyde for 10 min at room temperature. The nuclei were isolated and treated with 25 μl of 10× cutsmart buffer and 100 U of NlaIII (NEB, #R0125S), and incubated overnight at 37°C, 900 rpm to digest the chromatin. The next day, the restriction enzyme was inactivated by heat. The proximal, digested chromatin fragments were ligated by T4 DNA ligase (NEB, M0202) by incubating the reaction for at least 6 h at room temperature. After ligation, the DNA was reverse cross-linked and extracted by an equal volume of phenol:chloroform:isoamyl alcohol (Solarbio, P1013). The purified DNA was fragmented by sonication with Biorupter at high energy 30 s on, 60 s off, for two cycles. The fragmented DNA was examined by 1% DNA agarose gel electrophoresis. The size of the DNA product was ∼0.2–2 kb. To construct a 4C-seq library, a biotin-labeled primer targeting the region of interest was used for primer extension. The amplified DNA was purified by biotin–streptavidin pulldown. The biotin-labeled single-stranded DNA was ligated to an adapter by bridge linker-mediated ligation. The ligated DNA products were amplified by nested polymerase chain reaction (PCR), and 500–900 bp DNA products were purified by the Magen gel purification kit. The purified DNA was sequenced by Hiseq 4000 with 150 bp paired reads.

### Immunoprecipitation and western blotting

A total of 20 million native cells were used for immunoprecipitation experiments. A 1 ml aliquot of IP lysis buffer [20 mM Tris–HCl pH 7.5, 150 mM NaCl, 1% Triton X-100, sodium pyrophosphate, β-glycerophosphate, EDTA, leupeptin, phenylmethylsuflonyl fluoride (PMSF) and protein inhibitor cocktail] was used to resuspend cell pellets by rotating the cell lysates at 4°C for 30 min. The supernatant (5% cell lysis supernatant was saved as an input sample) was collected. Then, different antibodies were added to the pre-cleared whole-cell lysates and incubated overnight with rotation at 4°C. The protein–antibody complexes were incubated with Protein G for ∼2 h at 4°C. The beads were washed by sonification buffer four times and by high-salt buffer once. The supernatant was discarded, and a suitable volume of 1× SDS loading buffer was added. The eluted IP samples were boiled at 100°C for 5 min. The protein elution and input samples were used for western blotting analyses. Then the samples were run on different concentration SDS–polyacrylamide gel electrophoresis (PAGE) gels. They were transformed onto nitrocellulose membranes and the membranes were blocked with skim milk and then incubated with diluted primary antibody (1:1000–1:5000) overnight at 4°C. On the next day, the membranes were washed and incubated with secondary antibodies (1:5000). The membranes were washed and analyzed on a G.E AI 600 RGB imaging system.

### Live cell imaging

The cell lines that express proteins of interest with GFP tags were imaged on 35 mm glass-bottom dishes (MatTek, USA) by Nikon A1R-si. Before imaging, the cells were digested by 0.25% trypsin EDTA (Gibco, 25200-056) and transferred to the 35 mm glass-bottom dishes for ∼6 h. Then the cells were imaged by a confocal microscope (Nikon A1R-si). Hoechst 33342 (Beyotime, C1029) was used to stain the nucleus. Laser: 405 nm, 488 nm.

### ChIP-qPCR and ChIP-mass spectrometry

ChIP-quantitative PCR (qPCR) primers were designed and validated by NCBI primer-blast. The Ct value of input DNA was used for normalization. Calculation formula: %Input = 5%*2^ (Ct^Input^ – Ct^Sample^). The results of ChIP-qPCR were displayed by prism 8. At least two replicates were run for each qPCR. The statistical significance was evaluated by Student's *t*-test. The primers for the ChIP-qPCR can be found in Supplementary Table S9.

The suitable concentrations of SDS–PAGE protein gels to run ChIP protein preparations were chosen according to the molecular weight of target proteins. Cut gels of the full lane were used for MS analyses which were performed at the National Center for Protein Sciences at Peking University in Beijing, School of Life Science, Peking University) (Note: the part of the gels containing antibody was separated in to a single tube for MS analyses).

### DNA library construction

ChIP DNA libraries were constructed according to the previously reported TELP library construction protocol (68) or the NEBNext®Ultra™II DNA Library Prep Kit (NEB, E765S). Briefly, DNA was end-repaired, and a poly(C) tail was added to the purified end-repaired DNA products. Poly(C) tails were extended with biotin-labeled adapters. Biotin-labeled products were captured by streptavidin C1 beads (Invitrogen, 11206D). Quick ligase (NEB, M2200S) was used to ligate another adapter to the biotin-labeled products. The first-round PCR amplification was performed on beads. The second-round PCR amplification was carried out with the first-round PCR products. The final PCR products were run on a 2% DNA agarose gel. DNA products of 200–400 bp were purified by a Magen gel extraction kit. The NEB library construction was according to the NEBNext®Ultra™II DNA Library Prep Kit for Illumina® by following the manufacturer's instructions. The purified DNA was sequenced by Hiseq 4000 with 150 paired-end reads.

### RNA-sequencing

For RNA-seq, the ZBTB21-degron mESCs were induced by doxycycline for 24 h, and treated with IAA for 24 h. The untreated cells and IAA-treated degron cells were collected. RNA extraction was performed with Trizol by following the manufacturer's instructions. Our RNA-seq library was prepared by using the NEBNext® Ultra™ RNA Library Prep Kit for Illumina® (NEB, USA), which is regularly used in our lab (64,69,70). The libraries were prepared by following the manufacturer's instructions and sent to Novogene and sequenced on Illumina Hiseq 4000; 150 bp paired-end reads were obtained for downstream analyses.

### Chromatin fraction preparation

The chromatin was prepared by following the protocol published previously (65). A total of 10 million cells were incubated with NP-40 cell lysis buffer (10 mM Tris–HCl pH 8.0, 10 mM NaCl, 1.5 mM MgCl_2_, 0.01% NP-40 and protease inhibitors) to lyse cell membranes. The cell suspension was transferred to a 1.25 ml sucrose cushion [24% sucrose (w/v) in NP-40 cell lysis buffer] to extract the nuclei. The supernatant (cytoplasmic fraction) was collected. The nuclei were gently resuspended with 0.5 ml of glycerol buffer [20 mM Tris–HCl pH 8.0, 75 mM NaCl, 0.5 mM EDTA, 0.85 mM DTT, 50% (v/v) glycerol]. A 0.5 ml aliquot of nuclei lysis buffer (10 mM HEPES pH 7.6, 1 mM DTT, 7.5 mM MgCl_2_, 0.2 mM EDTA, 0.3 M NaCl, 1 M urea, 1% NP-40) was added to the resuspended nuclei. After centrifugation, the supernatant was saved as a nucleoplasmic fraction. The pellets were chromatin fractions used in this study; 1× SDS loading buffer was added to the chromatin pellets and boiled at 100°C for 5 min. Western blot was used to examine the target proteins.

### Chromatin RNA-seq (ChAR-seq)

For ChAR-seq, the ZBTB21-degron mESCs were induced by doxycycline for 24 h, and treated with IAA for 6 h. The chromatin was prepared by following the chromatin fraction preparation protocol as described above. The chromatin RNA was extracted from chromatin fractions with Trizol by following the manufacturer's instructions. The total RNA samples were firstly depleted of rRNAs and were constructed into next-generation sequencing libraries by following the experimental procedure for RNA-seq library preparation. The ChAR-seq libraries were sequenced in the same way as for RNA-seq libraries.

### ChIP-MS analysis

MS raw data were mapped to mouse or human proteins in the Uniprot database by the MS analysis platform in the National Center for Protein Sciences at Peking University, Beijing. Then, the peptide counts of all isoforms of a protein were combined to generate a single peptide–spectrum match (PSM) value for each protein. For mESCs, the candidate proteins were selected if the total peptide counts of the proteins in ChIP samples were at least twice more than those in input samples. For B16 and CH12F3 cells, the candidate proteins were identified by the R package DEP [*P*-value < 0.05, log2 fold change (FC) > 1] (71). The missing data were imputed using the ‘MinProb’ method. The candidate proteins were annotated according to previous publications (72,73), Uniport, Genecards and g:Profiler databases. In addition, the Gene Ontology (GO) determination of candidate proteins was carried out by using R package Cluster Profiler (74).

### ChIP-seq data analysis

Raw ChIP-seq reads were firstly trimmed using Cutadapt software (75) and then mapped to the genome using Bowtie2 software (76) in ‘–very-sensitive-local’ mode. ChIP-seqs that were performed in mESCs were mapped to the mm10 genome. ChIP-seqs that were performed in HEK293T cells were mapped to the hg19 genome. ChIP-seqs with spike-in control were mapped to both the mm10 and hg19 genome. Only uniquely and concordantly mapped reads were kept for further analysis. The mapped reads were converted to bigwig format using bamCoverage from deepTools2 software (77) in 10 bp bins with parameters ‘–normalizeUsing’ set to RPGC. The mapped reads from ChIP-seq with spike-in control in mESCs were converted to bigwig format normalized by the spike-in reads mapped to the hg19 genome. Peaks were called by MACS2 software (78) using a q threshold of 0.05. The two replicates were filtered by IDR software (https://github.com/nboley/idr) with an idr threshold of 0.05 to obtain repeatable peaks. Peaks that overlapped with the mm10 or hg19 blacklist were removed from the peak lists.

The genomic distribution of peaks was annotated according to the priority of promoters, enhancers, CTCF sites, gene bodies and others. The *cis-*regulated elements were defined as below:

Promoters: ±1 kb from transcription starting site (TSS) of Refseq genes.

Enhancers: H3K27ac peaks that did not overlap with promoters. Superenhancers were downloaded from a previous publication (79).

CTCF sites: CTCF peaks that did not overlap with enhancers and promoters.

Heatmaps and meta-gene profiles were calculated using computeMatrix of deepTools2 software centered around ±3 or ±5 kb of the peaks, SMC1 ChIA-PET anchors (*n* = 24 036, in Figure 2H) and genes. ChIP-seq signals of cohesin, NIPBL and ZBTB21 were counted at active gene promoters. The correlation curves between cohesin, NIPBL and ZBTB21 were fitted and smoothed by LOESS regression. Motifs surrounding ±250 bp of peak summits were predicted using Homer (http://homer.ucsd.edu/homer/ngs/index.html) software. The genes regulated by each peak were assigned by the R package ChIPseeker (80). GO analyses were performed using the R package ClusterProfiler (74).

To define ZBTB21-bound and unbound genes, genes were first filtered by requiring the existence of ZBTB21 peaks for the bound ones and no peaks for the unbound ones. Then the top 1000 genes were selected as bound genes based on the ChIP-seq signal of ZBTB21 at gene promoters. The bottom 1000 no peak genes were selected as the unbound genes. The mm10 gene set was obtained from the GENCODE database.

The differential cohesin peaks were first identified by the R package Diffbind using a false discovery rate (FDR) threshold of 0.05 to obtain more robust changes in 0–1, 0–6 and 1–6 h. These differential peaks were then merged and clustered using the R package hclust. For clustering analysis, a heatmap was used to visualize the changes of cohesin binding. Due to the high variability of cohesin binding signals at different regions, the z-score, which was calculated by (x – mean)/standard deviation (SD), was used to normalize the data in order to make the change patterns clearer. To determine the optimal cluster numbers, we used the ‘Elbow method’ to test a range of cluster numbers and finally selected three clusters to reflect the biologically meaningful changes.

Peaks of ZBTB21, cohesin and CTCF ChIP-seq datasets in Figure 2C were first merged and then clustered by their ChIP-seq signal using the R package k-means. The control peaks were randomly selected from the mm10 genome (excluding the merged peak regions) to the same number of ZBTB21 peaks. ChIP-seq densities of cohesin, RAD21, NIPBL, WAPL and CTCF were then plotted according to the clustered and control categories. The heatmap in Figure 6C was generated using the same method utilizing the merged peaks of the five ZBTB transcription factors, cohesin and CTCF. The 1/2/3/4+ ZBTB peaks in Figure 6F were defined as the regions co-occupied by one, two, three, four and more than four ZBTB transcription factors.

Statistical tests used in this study were all determined by two-side Wilcoxon test or Student's test as mentioned in each figure legend.

### Hi-C data analysis

The bridge linkers of raw Hi-C reads were first trimmed using trimLinker of the ChIA-PET2 software (81). The processed reads were then mapped to the mm10 genome and filtered to obtain valid contact pairs using the HiC-Pro pipeline (82). RCP duplicated, dangling ended, re-ligated and self-circled read pairs were discarded for further analysis. Long-range (>20 kb) intrachromosomal valid pairs of all the samples were randomly sampled to the same depth (*n* = 34 M) and merged together between the two replicates (*n* = 68 M) to obtain higher resolution. The resolution of our Hi-C library is ∼25 kb, so the interactions <20 kb are below our Hi-C resolution, which are potential false-positive interactions. Therefore, we removed the interactions <20 kb in our analyses. Many previous publications also removed the short-distance interactions for subsequent analysis (83–85). The merged read pairs were converted to ‘.hic’ format using hicpro2juicebox in HiC-Pro software and then converted to ‘.cool’ format using hic2cool software (https://github.com/4dn-dcic/hic2cool). The ‘.cool’ format Hi-C maps were balanced by cooler software (86). The snapshots of contact maps were visualized using Juicebox software (87) after Knight–Ruiz (KR, also known as balanced) normalization. The Hi-C maps were iteratively corrected using the ‘balance’ command from cooler software.

A/B compartments were called using call-compartments of Cooltools software (https://github.com/open2c/cooltools) and adjusted by H3K27ac ChIP-seq signals. The saddle plots were computed by compute-saddle of Cooltools software at 50 kb resolution and normalized by the expected matrix in ‘–quantiles’ mode. The enrichment score was calculated as (AA + BB)/2AB, where AA refers to the mean normalized density of bins in the top 20% eigenvector scores, BB refers to the mean normalized density of bins in the bottom 20% eigenvector scores, and AB refers to the mean normalized density of bins between AA and BB.

To plot meta-TAD, we firstly calculated the average contact probability for all loci at a certain distance as the expected Hi-C contact value for each chromosome. Then we transformed the KR-normalized Hi-C matrix into an observed/expected matrix by dividing each normalized observed value by its corresponding expected value at that distance. Second, we carried out a 2D meta feature analysis by piling up individual submatrices into an average matrix using Coolpup software (v0.9.5) with ‘–rescale’ and ‘–local’ parameters. In general, this is similar to meta-gene analysis, commonly performed for ChIP-seq data. The positions of TADs were downloaded from Bonev *et al.* (83). Each TAD was padded by the neighboring regions with the same length as the TAD. Hi-C O/E maps at 25 kb resolution were used for the analysis.

Loops were called in 5, 10 and 25 kb resolution using Mustache software (88) with parameters ‘-pt’ and ‘-st’ set to 0.1 and 0.88. Loops of different resolutions were extended to the lowest resolution (25 kb), and merged together for further analysis. Loops from untreated and IAA-treated samples were first merged together and then ranked and plotted by their loop length in Figure 4D. The color of each loop indicates the log2FC of loop strength before and after ZBTB21 degradation. The gene set enrichment analysis (GSEA) in Figure 4E indicates that IAA-induced loops (the loops that occurred after ZBTB21 degradation) distribute at the top longest loops among all the merged loops. Loops were ranked by their loop length and marked in red if they were hits in IAA-gained loop sets. The less responsive loops (see below) serve as negative controls. Statistics were computed by permuting the loop labels and recomputing the enrichment score of the IAA-induced or less responsive loop sets to generate a null hypothesis (89). Local Hi-C interactions of promoters, enhancers, CTCF sites and superenhancers were calculated by Coolpup software at 25 kb resolution and normalized by the expected matrix. The interaction frequency enrichment curves were fitted using all the bins that interacted with the bin containing the peaks.

ZBTB21-responsive loops were defined as the top 500 loops that contained both ZBTB21 peaks and down-regulated cohesin peaks; less ZBTB21-responsive loops were defined as the bottom 500 loops that did not contain ZBTB21 peaks and down-regulated cohesin peaks. Loops were ranked by the sum of the ZBTB21 ChIP-seq signal and the absolute value of log2FC of cohesin ChIP-seq signal upon ZBTB21 degradation at the ATAC-Seq peaks inside each loop. Because the loop length distribution analyses indicated that loops >500 kb showed more changes, loops >500 kb were classified into ZBTB21-responsive and less-responsive loops. Down-regulated cohesin peaks were identified using cohesin ChIP-seqs before and after ZBTB21 degradation for 6 h by Diffbind software using a *P*-threshold of 0.05. ZBTB21, differential cohesin and GRO-seq signals were plotted by heatmaps centered around the ATAC-seq peaks in ZBTB21-responsive (*n* = 7973) and less-responsive (*n* = 1112) loops. GRO-seq signals were not classified into + or – strands due to the inability to distinguish ATAC-seq peaks between the + or – strands. APA plots of loops were calculated by Coolpup software at 25 kb resolution and normalized by the expected matrix. The enrichment score was computed as the mean normalized density of the central 3*3 bins.

The genes were assigned to ZBTB21-responsive and less-responsive loops and plotted using the absolute value of log2 ChAR-seq fold change upon ZBTB21 degradation for 6 h (Figure 5D). The enrichment of down-regulated, unchanged and up-regulated genes was shown as an observed/expected ratio in the same way as in a previous publication (90). The percentages of genes in each category of loops (observed) were normalized by the genome-wide percentage of the genes in each category (expected). The red dots indicate the observed/expected ratio of ZBTB21-responsive and less-responsive loops. ZBTB21-responsive and less-responsive loops were randomly permuted 10 000 times across the genome to generate distributions of the ratio (the violin plot), and significance was determined by the distributions. The insulation scores of the ice-normalized Hi-C matrix of ZBTB21 were computed by cworld software (https://github.com/dekkerlab/cworld-dekker) at 25 kb resolution. The insulation scores of the CTCF Hi-C matrix were computed by cworld software at 20 kb resolution. CTCF Hi-C data were downloaded from a previous publication (25).

### 4C data analysis

Raw 4C reads were first filtered and trimmed by the enzyme sites using trimLinker from ChIA-PET2 (81) software. The paired-end reads were selected for downstream analysis by Cutadapt (75) software if the 5′ end of reads1 contains bait sites. The processed reads2 were then mapped to the mm10 genome by bowtie2 (76) software using ‘–very-sensitive-local’ parameters. PCR duplicates, self-religations and reads longer than the library size were discarded. The bigwig files were generated by bamCoverage of deepTools (91) software and normalized to the 1× coverage of the mm10 genome using ‘–binSize 10 –normalizeUsing RPGC’ parameters.

For displaying 4C track, we smoothed the signals in the same way as in a previous publication (41), i.e. the genome to be displayed was binned using a 50 bp sliding window. At each bin, the smoothed signal was calculated as the mean signal of a 5 kb region around the bin. For 4C quantification, the signal at a specific region (chr8:88940000–89060000) was normalized by the signal of a 4 Mb downstream background region (chr8:84940000–85060000) using the non-smoothed normalized signals.

### RNA-seq data analysis

Raw RNA-seq reads were first trimmed using Cutadapt software and then mapped to the mm10 genome using STAR software (92). Primarily mapped reads without PCR duplication were kept for further analysis. The signals of genes downloaded from GENCODE vM25 were obtained using featureCounts software. Differentially expressed genes (DEGs) were identified by the R package DEseq2 (93) with an FDR threshold of 0.05.

### ChAR-seq data analysis

Raw ChAR-seq reads were first trimmed using Cutadapt software and then mapped to the mm10 and dm6 genome using bowtie2 software (v2.3.5.1). Uniquely mapped reads without PCR duplication were kept for further analysis. The expression of the mm10 genes was counted by featureCounts software at gene body regions [+300 from the TSS to the transcription end site (TES)] and normalized by the spike-in reads mapped to the dm6 genome in each sample. DEGs were identified by the R package DEseq2 (93) with an FDR threshold of 0.05.

### Distribution percentage, CRISPR score and expression analysis

ChIP-seq peaks of transcription factors in Supplementary Figure S1 were downloaded from the ENCODE and Cistrome databases (Supplementary Table S8). ChIP-seqs of H3K27ac and CTCF in different cell lines, as well as genes in the mm9/mm10 and hg19/hg38 genome, were downloaded to generate the genomic regions of promoters, enhancers and CTCF sites in corresponding cell lines. Peaks were annotated as these *cis-*elements and clustered based on the percentage of occupancy using the R package hclust. Gene expression data of the transcription factors in 53 different tissues were obtained from the GTEx database. All the samples were grouped and averaged by tissues. The expression percentage of each gene was defined as the percentage of tissues whose averaged transcripts per million (TPM) value was >10. CRISPR scores of human genes were obtained from a previous publication (94), which were computed by log2FC in abundance of each sgRNA between the initial and final populations. Genes were ranked by their CRISPR scores and defined as essential if their CRISPR scores were <0 and the *P*-value was <0.05.

## RESULTS

### Cohesin ChIP-MS identifies known and potential regulators of 3D chromatin organization

**Figure 1.**
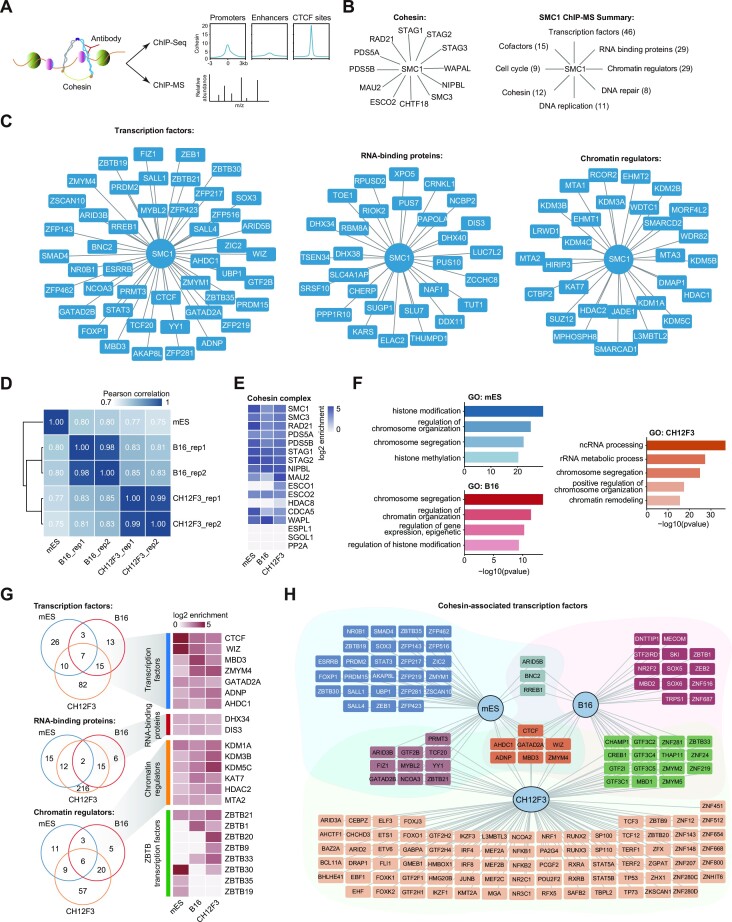
Cohesin ChIP-MS identifies candidate regulators of 3D chromatin organization. (**A**) Schematic representation of the cohesin chromatin proteomic profiling (ChIP-MS) procedure. Formaldehyde-cross-linked chromatin was sonicated, and ChIP was performed with specific antibodies (SMC1). The ChIP-purified DNA was subjected to DNA sequencing, and the ChIP-purified proteins were subjected to MS analyses. Meta-gene results showing the occupancy of cohesin (SMC1) in mESCs are presented in the right panel. (**B**) Cytoscape analysis of the cohesin ChIP-MS-identified cohesin components (left panel) and a summary of the categories of the identified proteins (right panel). The numbers within parentheses represent the numbers of identified proteins in each category identified by cohesin ChIP-MS. (**C**) Cytoscape analysis of cohesin ChIP-MS-identified transcription factors, RNA-binding proteins and chromatin regulators in mESCs. (**D**) The heatmap shows the Pearson correlation coefficients between each of the samples of mES, B16 and CH12F3 cells. The coefficients are clustered by hierarchical clustering. (**E**) The heatmap shows the log2 enrichment of the PSM values of cohesin components in ChIP samples over the input sample in mES, B16 and CH12F3 cells. (**F**) GO enrichment terms of the enriched proteins identified by cohesin ChIP-MS in mES, B16 and CH12F3 cells. (**G**) Venn diagram showing the shared transcription factors, RNA-binding proteins and chromatin regulators identified by cohesin ChIP-MS in mES, B16 and CH12F3 cell lines. The heatmap (right) shows the log2 enrichment of the PSM values of shared proteins and ZBTB transcription factors in ChIP samples over the input sample. (**H**) Network showing the transcription factors preferentially enriched in mES, B16 and CH12F3 cell lines. The transcription factors that were statistically significantly enriched are shown in the plot (*P*-value < 0.05, log2FC > 1). Transcription factors within different color backgrounds indicate that the proteins were detected in a specific cell line or different cell lines.

To comprehensively identify protein factors that contribute to 3D genome organization, we performed cohesin ChIP-MS in mESCs (Figure 1A) because cohesin plays a broad role in chromatin architecture (84,95). Cohesin ChIP-MS identified almost all known components and direct regulators of cohesin complexes (Figure 1B). The genomic distribution revealed by cohesin ChIP-seq among enhancers, promoters and CTCF sites was consistent with that obtained in previous studies (31–33) (Figure 1A; Supplementary Table S1). These results indicated the success of our cohesin ChIP experiment. The identified proteins were then grouped into different categories according to the UniProt, GeneCards and PubMed databases (Figure 1B; Supplementary Table S2; see also the Materials and Methods). Many known chromatin organization regulators were identified in our cohesin ChIP-MS data; for example, the known chromatin architecture proteins CTCF, YY1, ZFP143, ADNP and WIZ were among the transcription factors identified by cohesin ChIP-MS (Figure 1C) (25,41–44,47). The exosome complex was predicted to regulate superenhancer chromosomal interactions (96,97). The exosome component DIS3 was among the RNA-binding proteins identified by cohesin ChIP-MS (Figure 1C). Together, these analyses suggest that our cohesin ChIP-MS data in mESCs are reliable.

To provide further information on cohesin-interacting proteins, we performed genomic localization, expression specificity and essential score analyses. Genomic localization is expected to reveal the types of chromatin interactions regulated by these proteins, but not all these proteins have reliable ChIP-seq datasets in mESCs. We therefore searched the ChIP-seq datasets for cohesin-associated proteins from different cell lines included in the ENCODE and Cistrome databases, and calculated the occupancy percentages of promoter-, enhancer- and CTCF-binding regions in the corresponding cells (Supplementary Figure S1A). We performed gene expression analyses across different tissues by using gene expression datasets from the GTEx database (Supplementary Figure S1B) and conducted CRISPR score analyses to evaluate the essentiality of the cohesin-associated proteins (Supplementary Figure S1C) (94). We hypothesized that constitutively expressed, essential chromatin structural proteins, such as CTCF and YY1, play a general role in 3D genome organization (25,41,42), while less essential specifically expressed proteins, such as ADNP and AP-1, play a cell type-specific role or regulate a subset of chromatin interactions (44,48). Although these results were generated from different cells, they integrate useful information for investigating these cohesin interactors in the future.

It would be informative to compare our cohesin ChIP-MS data with the cohesin interactome in different cell types. Previous studies have identified the cohesin interactome in human HCT116 and MOLT-4 cells (98,99). However, these MS data are not comparable with our data, as they were generated by different experimental protocols and in different species. We therefore performed cohesin ChIP-MS analysis in mouse B16 cells from skin and CH12F3 B lymphocytes by using a similar approach to the one we used for mESCs. We used the R package DEP (Differential Enrichment analysis of Proteomics data) to perform statistical analyses of these MS data (*P*-value < 0.05, log2FC > 1) and identified 260 and 1332 cohesin-interacting proteins in B16 and CH12F3 cells, respectively (the number of interacting proteins was quite different, possibly due to the different efficacy/accuracy of cohesin ChIP-MS in different cells). Our cohesin ChIP-MS data were quite reproducible and were strongly enriched in known cohesin components (Figures 1D, E), indicating that the quality of these MS data was good. We further compared the cohesin ChIP-MS data of B16 cells and CH12F3 cells with the cohesin ChIP-MS data of mESCs. GO analysis showed that the factors identified by cohesin ChIP-MS were mainly enriched in chromosome organization, histone modification and non-coding RNA processing terms in different cell types (Figure 1F). The factors that have previously been demonstrated to regulate cohesin, namely CTCF, WIZ and ADNP, were identified in three different cohesin ChIP-MS preparations (25,41–44,47). In addition to those known cohesin-interacting proteins, additional transcription factors, RNA-binding proteins and chromatin regulators were also detected in the cohesin ChIP-MS preparations (Figure 1G). Moreover, many transcription factors were preferentially detected in different cell lines (Figure 1H), which may be candidate regulators of cell type-specific 3D chromatin structures. These analyses provide a resource listing constitutive and cell type-specific cohesin-interacting proteins in different cell types.

Many of the proteins identified by cohesin ChIP-MS have not previously been reported to play roles in chromatin organization or to function together with cohesin. We detected four ZBTB transcription factors, ZBTB19, ZBTB21, ZBTB30 and ZBTB35, in mESCs (Figure 1C, G), which were of considerable interest to us because ZBTB transcription factors are known to regulate insulator activities, as suggested by the functions of ZBTB factors in *Drosophila* (52–56). Insulator function has been proposed to involve determining 3D chromatin organization (4,100,101). Since ZBTB21 was detected in cohesin ChIP-MS preparations in three different cell types (Figure 1G), we primarily investigated the molecular functions of ZBTB21 in this study.

### ZBTB21 interacts with cohesin at active promoters

**Figure 2.**
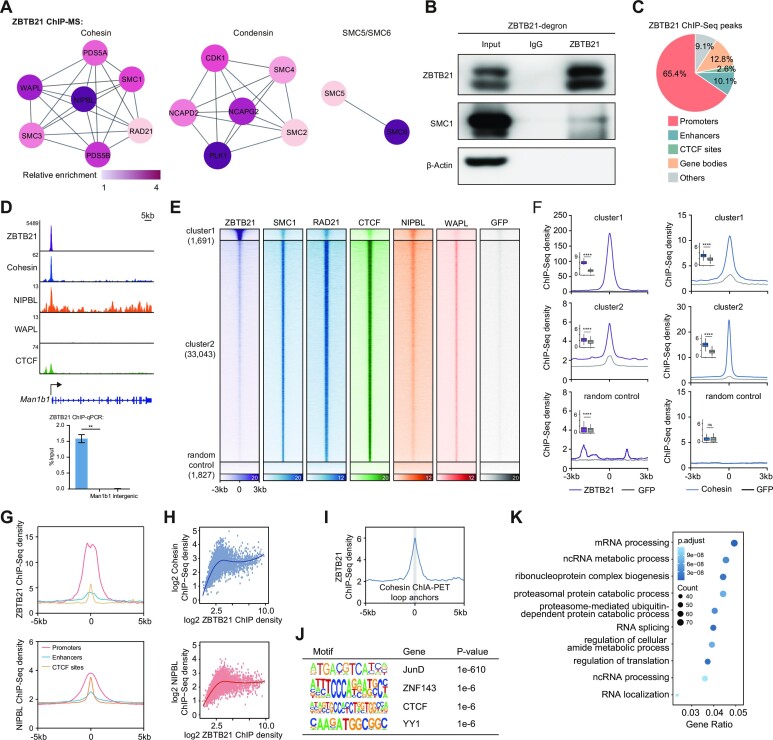
ZBTB21 interacts with cohesin at gene promoters. (**A**) Networks summarizing the SMC complex identified by ZBTB21 ChIP-MS in mESCs. Lines represent interactions with different proteins in the STRING database. The ZBTB21-associated proteins detected by ChIP-MS were identified according to the following cut-off criteria: unique peptides ≥ 2, (ZBTB21-IP)/(GFP-IP) ≥2. The definition of relative enrichment is described in the Materials and Methods. (**B**) Western blotting analyses of the ZBTB21 IP products in native ZBTB21-degron–GFP mESC extracts. A GFP antibody was used for the IP of ZBTB21-degron–GFP. IgG and β-actin served as negative controls. SMC1 was also detected. Note: there are two bands for ZBTB21 in the western blotting analyses, which may be due to the splicing isoforms. (**C**) Genomic distribution of ZBTB21 ChIP-seq peaks in promoters, enhancers, CTCF sites, gene bodies and other regions in mESCs. Each peak of ZBTB21 was assigned to a genomic feature, and the proportions of the features are shown as a pie chart. Note: the genomic binding of ZBTB21 shown in Supplementary Figure S1A is based on publicly available data generated in 293T cells. (**D**) ChIP-seq snapshots of ZBTB21, cohesin, NIPBL, WAPL and CTCF at the Man1b1 locus. ChIP-qPCR validation of ZBTB21 ChIP-seq signals in Man1b1 promoter regions is shown at the bottom. Two replicates were performed for each site. Statistical significance was evaluated by Student's *t*-test (**P < 0.01). The error bars represent SDs. The information for the qPCR primers is listed in Supplementary Table S9. (**E**) Heatmap showing the ChIP-seq densities of ZBTB21, SMC1, RAD21, CTCF, NIBPL, WAPL and GFP among merged peaks of ZBTB21, cohesin and CTCF. The peaks of ZBTB21, cohesin and CTCF were first merged together and then clustered using their ChIP-seq signals by the k-means method. The control peaks were randomly selected from the mm10 genome (excluding the merged peak regions) to the same number of ZBTB21 peaks (*n* = 1827). GFP ChIP-seq was performed with an anti-GFP antibody using wild-type mESCs, which served as a negative control for ZBTB21. ChIP-seq data of cohesin (SMC1), RAD21 and WAPL were downloaded from previous publications (33,90,127). (**F**) Meta-plots displaying ZBTB21 (left panel), cohesin (right panel) and GFP ChIP-seq signals in the different clusters. The lines indicate the mean ChIP-seq signal of ZBTB21, cohesin and GFP in each cluster. The boxplots (insert) indicate the log2 densities of ChIP-seq signals. Significance was determined by Wilcoxon test (****P < 0.0001). (**G**) Aggregated plots showing the mean occupancy of ZBTB21 and NIPBL ChIP-seq signals at ±5 kb around the centers of promoters, enhancers and CTCF sites. (**H**) Scatter plots showing the correlation between ZBTB21 ChIP-seq density and cohesin/NIPBL ChIP-seq density among active gene promoters. The curve was fitted and smoothed by LOESS regression. (**I**) Meta-illustration of ZBTB21 ChIP-seq signals at ±5 kb around SMC1 ChIA-PET loop anchors [*n* = 24 036, generated from a previous publication (95)]. (**J**) Table showing the representative enriched motifs of known chromatin structure proteins at ZBTB21 ChIP-seq peaks. Motif analysis was performed with all ZBTB21 binding peaks. (**K**) GO enrichment terms of ZBTB21 ChIP-seq-bound genes identified by ClusterProfiler. The gene ratio is the ratio of the number of genes associated with each specific GO term divided by the total number of ZBTB21-bound genes. The color bar indicates the adjusted *P*-value. Circle size indicates the number of enriched genes.

ZBTB21 ChIP-MS and ChIP-seq were performed to validate the associations between ZBTB21 and cohesin. We first introduced a degron–GFP tag into the C-terminus of endogenous ZBTB21 in mESCs (Supplementary Figure S2A). Genotyping analyses confirmed the success of homozygous knockin (Supplementary Figure S2B). Then, widely used GFP antibodies were chosen to perform ChIP-MS and ChIP-seq in ZBTB21-degron–GFP cells. Consistent with the cohesin ChIP-MS results, cohesin components were preferentially identified in ZBTB21 ChIP-MS preparations (Figure 2A; Supplementary Table S2). Interestingly, condensin and SMC5/SMC6 components were also detected in ZBTB21 ChIP-MS preparations (Figure 2A). The protein–protein interactions between ZBTB21 and cohesin were further validated through IP–western blotting analyses of native cell lysates but not IgG IP preparations (Figure 2B), which served as a negative control. To gain deeper insight into ZBTB21-mediated cohesin regulation, we performed ZBTB21 immunoprecipitation after ZBTB21 depletion or treatment with different nucleases. The results showed that ZBTB21 forms specific protein−protein interactions with cohesin (Figures S2C, D). The genomic localization of ZBTB21 was carefully characterized to obtain functional insights into ZBTB21-mediated regulation. Meta-gene analysis and a pie chart displaying the genomic distribution of the ZBTB21 peaks indicated that ZBTB21 preferentially bound gene promoters (Figure 2C). We identified ZBTB21 chromatin-binding sites in mESCs (Supplementary Tables S1 and S3). These binding sites could be independently validated by ChIP-qPCR (Figure 2D; Supplementary Figure S2E). Furthermore, genomic localization analyses indicated that ZBTB21-occupied regions showed weak binding of cohesin and the cohesin loading factor NIPBL, which was significant compared with the signals in randomly selected regions and GFP ChIP-seq signals (Figure 2E). ZBTB21 peaks belonged to Cluster 1 and were associated with weak cohesin ChIP-seq signals. Cluster 2 did not have ZBTB21 peaks and was associated with strong cohesin binding signals (Figure 2F).

Furthermore, ZBTB21 and NIPBL preferentially occupy the gene promoters (Figure 2G). As the ZBTB21 ChIP-seq density increased, the chromatin binding strength of cohesin and NIPBL at gene promoters first increased and was then maintained at a fairly consistent level (Figure 2H). ZBTB21-binding sites were enriched at the cohesin loop anchors identified by cohesin ChIA-PET (Figure 2I). Motif analyses of the ZBTB21 ChIP-seq peaks revealed the enrichment of transcription factor [e.g. JUND (a component of the AP-1 family), ZNF143, CTCF and YY1] functions in 3D chromatin organization (Figure 2J). GO analyses of the ZBTB21-bound gene indicated that these genes were involved in RNA metabolism pathways (Figure 2K). These results suggested that ZBTB21 was associated with cohesin chromatin binding at promoters, indicating potential involvement in cohesin promoter occupancy.

### ZBTB21 depletion leads to decreased chromatin binding of cohesin

**Figure 3.**
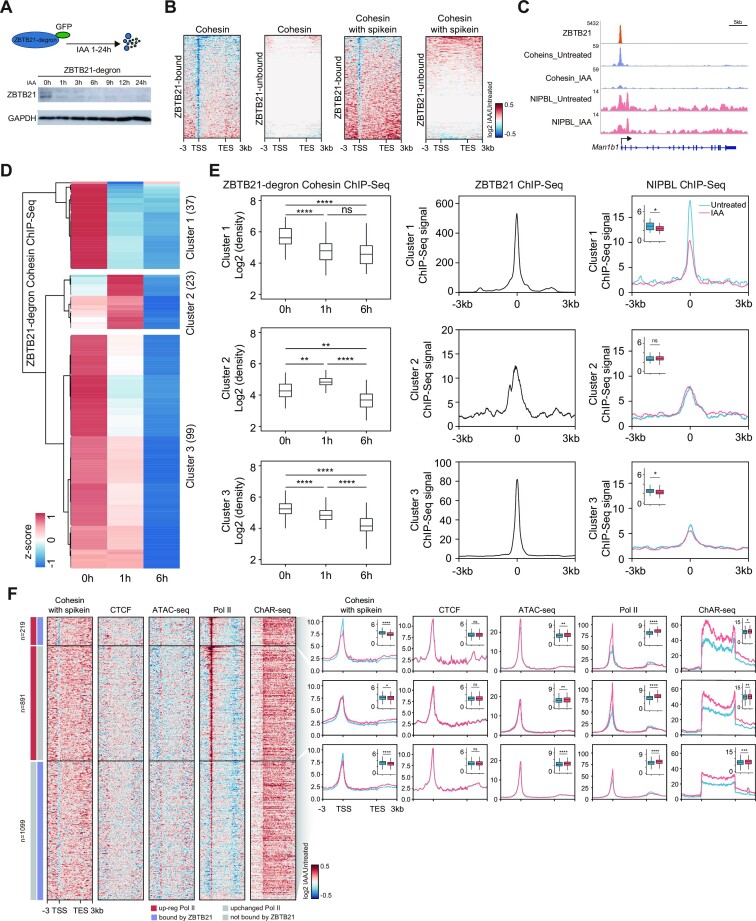
ZBTB21 depletion leads to decreased chromatin binding of cohesin. (**A**) Western blotting analysis of ZBTB21 degradation in ZBTB21-degron–GFP mESCs after IAA treatment at different time points. ZBTB21 was identified with a GFP antibody because it was endogenously GFP tagged, and GAPDH served as a loading control. (**B**) Heatmap showing the occupancy changes of cohesin with or without spike-in controls at ZBTB21-bound and ZBTB21-unbound genes. Each line of the heatmap indicates the log2FC in the ChIP-seq signal at a gene and its ±3 kb flanking region. The color bar indicates the log2FC in ChIP-seq signals. For the definition of ZBTB21-bound and unbound genes, see the Materials and Methods. (**C**) ChIP-seq snapshots of the ZBTB21 signal and down-regulated cohesin and NIPBL signals upon ZBTB21 degradation at the Man1b1 locus. (**D**) Hierarchical clustering of cohesin ChIP-seq density in peaks that were differentially regulated at 0–1, 0–6 or 1–6 h. The differentially regulated peaks (FDR < 0.05) were identified by DiffBind, merged together and classified into three clusters (see the Materials and Methods). (**E**) Left: boxplot showing the log2 cohesin ChIP-seq density of differentially regulated peaks in each cluster. Middle: meta-analyses displaying the mean ZBTB21 ChIP-seq densities at differentially regulated cohesin peaks in each cluster. Right: meta-analyses displaying the mean NIPBL ChIP-seq signals at differential cohesin peaks in each cluster after ZBTB21 degradation for 1 h. The inserts indicate the log2 densities of ChIP-seq signals. Significance was determined by the Wilcoxon test (****P < 0.0001; **P < 0.01; *P < 0.05; ns, not significant). (**F**) Heatmap showing RNA polymerase II (Pol II), cohesin, CTCF ChIP-seq, ATAC-seq and ChAR-seq signals among the genes with up-regulated Pol II (red bar) and ZBTB21 binding (purple bar) (*n* = 219), genes with up-regulated Pol II and without ZBTB21 binding (*n* = 891) and genes with less changed Pol II (below our cut-off) and ZBTB21 binding (*n* = 1099) (left). The color bar indicates the log2FC in ChIP-seq, ATAC-seq or ChAR-seq signals. Genes with up-regulated Pol II binding were identified by the R package DiffBind (FDR < 0.05). The meta-plots of the three clusters of genes are plotted on the right. The boxplot (insert) shows the changes in the ChIP-seq, ATAC-seq signals at ±100 bp around the TSS, or ChAR-seq expression changes upon ZBTB21 degradation. Significance was determined by the Wilcoxon test (****P < 0.0001, ***P < 0.001, **P < 0.01, *P < 0.05).

To obtain functional insights into the interactions between ZBTB21 and cohesin, we performed cohesin (SMC1) and NIPBL ChIP-seq after ZBTB21 depletion (Suppelementary Tables S1 and S4). The western blotting results confirmed the acute depletion of ZBTB21 proteins in our ZBTB21-degron mESCs (Figure 3A). We also performed cohesin ChIP-seq with equal amounts of *Drosophila* cells spiked-in. The tornado plot displaying the spike-in and non-spike-in cohesin ChIP-seq signal changes at the ZBTB21-bound and unbound promoters consistently showed that ZBTB21 depletion decreased cohesin binding at ZBTB21-bound promoters but not for the unbound promoters (Figure 3B). The chromatin binding of NIBPL at ZBTB21-bound gene promoters also displayed a weak decrease (Supplementary Figure S2F). Screenshots of specific genes showed that ZBTB21 depletion led to decreased cohesin and NIPBL binding at the Man1b1, Mmab and Znrd1 gene promoters (Supplementary Figure S2G). We also performed cohesin ChIP after ZBTB21 depletion, and ZBTB21 resumed after withdrawing the IAA. The results showed that cohesin chromatin binding decreased after ZBTB21 depletion and then resumed after the withdrawal of IAA (Supplementary Figure S2H), suggesting that the roles of ZBTB21 in cohesin chromatin binding are specific. Collectively, these results support that ZBTB21 specifically regulates cohesin chromatin occupancy, even though direct protein−protein interactions with recombinant proteins are currently lacking.

We next carried out time-course cohesin ChIP-seq after ZBTB21 depletion to determine the dynamic effects on cohesin. The clustering analyses of the differential peaks indicated the existence of three clusters (Figure 3D, E): in Cluster 1, the cohesin signals rapidly decreased, and the peaks displayed high-density ZBTB21 signals; in Cluster 2, the cohesin signals first increased and then decreased, and the peaks displayed less ZBTB21 binding; in Cluster 3, the cohesin signals gradually decreased, and the peaks displayed an intermediate level of ZBTB21 binding. Moreover, there was an obvious decrease in NIPBL binding in Cluster 1, a small but significant decrease in Cluster 3 and no significant change in Cluster 2 after the rapid depletion of ZBTB21 (Figure 3E). We then performed Pol II and CTCF ChIP-seq and ATAC-seq to obtain functional insights into ZBTB21-mediated cohesin regulation (Figure 3F). The results revealed that ZBTB21 depletion leads to global transcriptional activation, as shown by the increased signals of Pol II ChIP-seq and ChAR-seq, suggesting that ZBTB21 functions in transcription repression, which is consistent with the findings of a previous study (102), but could not simply explain its effects on cohesin chromatin binding. The ATAC-seq signals and CTCF chromatin binding did not show obvious changes after ZBTB21 depletion, indicating that CTCF and chromatin accessibility also could not explain ZBTB21-mediated cohesin binding.

To gain functional insights into ZBTB21 chromatin binding, we performed total RNA-seq and ChAR-seq to enrich nascent RNAs after ZBTB21 depletion (Supplementary Table S5). The differential analyses showed that 473 DEGs in ChAR-seq have ZBTB21 binding, but those are a subset of ZBTB21-bound genes. The number of those genes was higher than the number of genes with similar RNA-seq results (Supplementary Figure S2I). These results are consistent with a recent finding that there is a poor correlation between transcription factor binding and acutely affected gene expression (103). We also examined the expression changes of subunits of cohesin, condensin and SMC5/6, and found that the expression of most of the subunits did not change. Some of the subunits showed up-regulation, but less than our cut-off (Supplementary Figure S2J; Supplementary Table S5). However, we could not completely rule out other secondary effects of ZBTB21-mediated transcriptional repression that might contribute to the reduction in cohesin binding.

### ZBTB21 contributes to 3D chromatin interactions

**Figure 4.**
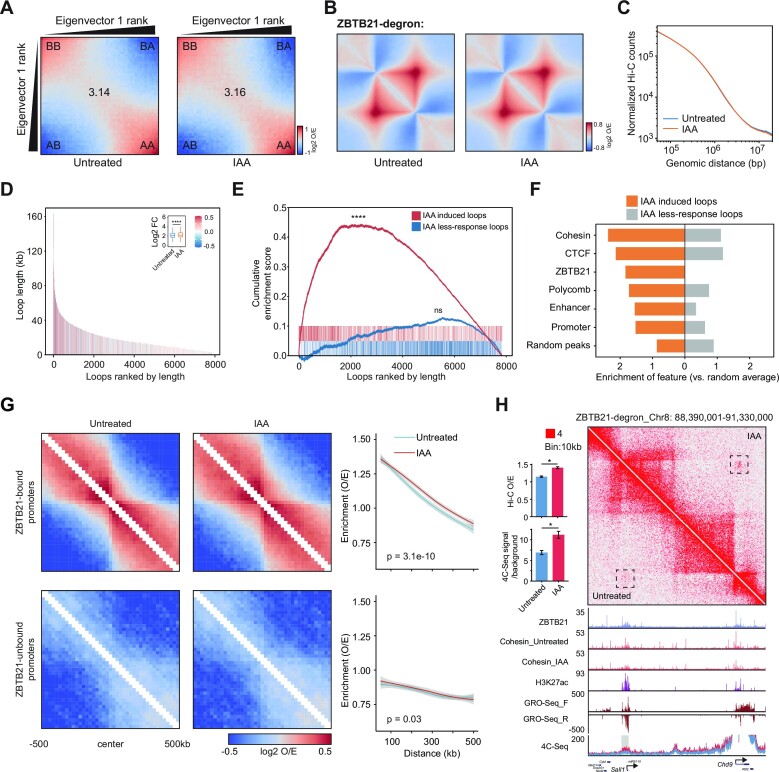
ZBTB21 insulates small-scale 3D chromatin interactions. (**A**) Saddle plot showing the change in compartmentalization strength upon ZBTB21 degradation. The enrichment score was calculated as (AA + BB)/2AB. (**B**) Meta-TAD analysis showing that the TAD strength does not change upon ZBTB21 degradation. The genomic positions of TADs were downloaded from Bonev *et al.* (83). (**C**) Hi-C enrichment counts at different genomic distances before and after ZBTB21 degradation generated by HiCExplorer. The *x*-axis starts from 50 kb. Hi-C maps with 50 kb resolution were used for the plot. (**D**) Changes in loop strength upon ZBTB21 degradation. The loops from untreated and IAA samples were first merged and then ranked by their loop strength. The *x*-axis indicates the rank of the loops, the *y*-axis indicates the loop length and the color indicates the log2FC in loop strength. The boxplot (inset) shows the changes in loop strength. Significance was determined by the Wilcoxon test (****P < 0.0001). (**E**) GSEA indicated that the gained loops in IAA samples were distributed at the top of all the merged loops ranked by length (see the Materials and Methods). The less responsive loops are shown as a negative control. Statistics were computed by permuting the loop labels and recomputing the enrichment score of the IAA-induced or less-responsive loop sets to generate a null hypothesis (89). (**F**) Enrichment of ChIP-seq peaks at anchors of induced loops that arise after ZBTB21 degradation and loops less responsive to ZBTB21. The enrichment of the peaks was calculated using the number of ChIP-seq peaks of the *cis-*regulatory elements inside the loops normalized by random averaging. Random averaging was defined by permuting the loops across the genome 1000 times and calculating the average number of ChIP-seq peaks at these random loop anchors. (**G**) Aggregate target-centered Hi-C interaction maps showing the changes in contact strength upon ZBTB21 degradation around ZBTB21-bound and ZBTB21-unbound promoters. Pile-up maps were plotted at a 25 kb resolution and normalized by the expected matrix. The color bar indicates the log2 ratio of observed/expected density. The right panel shows the decay curve of the Hi-C interaction frequencies extracted from the observed/expected matrix in the corresponding regions. Significance was determined by the Wilcoxon test in paired mode. (**H**) Hi-C contact maps for the regions of chromosome 8 (88–91 Mb) at a 10 kb resolution in untreated and IAA-treated (6 h) ZBTB21-degron cell lines. Genome browser snapshots of ZBTB21, cohesin (SMC1) and H3K27ac ChIP-seq signals, GRO-seq (GRO-seq_F for signals of the sense strand, GRO-seq_R for signals of the antisense strand) and 4C-seq signals. A subset of active genes is shown. The quantification of increased regions (indicated by a dashed rectangle or gray bar) of Hi-C (top) and 4C-seq signals (bottom) before and after ZBTB21 degradation are displayed in the left panel. Significance was determined by Student's *t*-test (*P < 0.05). The error bars indicate the SD. The red rectangle indicates the normalized Hi-C contact frequency.

To explore the direct roles of ZBTB21 in 3D chromatin organization, we performed BAT Hi-C after ZBTB21 depletion for 6 h. The BAT Hi-C method, which was previously developed by our laboratory (64,66), can generate high-resolution chromatin interaction maps in a more economical way than the classical *in situ* Hi-C method. The Hi-C data were processed via previously established pipelines (64,66). The mapping rate was high and was quite reproducible between the two replicates (Supplementary Figure S3A; Supplementary Tables S1 and S6). The saddle plot and meta-TAD analyses indicated that ZBTB21 depletion did not obviously affect large-scale chromatin interactions in mESCs, such as A/B compartments and TADs (Figures 4A, B), and the decay curve of Hi-C interaction frequencies across different genomic distances likewise indicated no change (Figure 4C), suggesting that ZBTB21 was not required for global, large-scale chromatin interactions in mESCs.

Chromatin loops were then compared before and after ZBTB21 depletion with IAA. Chromatin loops were identified with Mustache software (88), and Hiccups produced results with a similar trend. There were 5606 loops identified in the untreated condition and 6423 loops identified under IAA treatment. Overlap analyses indicated that 2867 loops were induced after ZBTB21 depletion. Density distribution analyses and loop strength changes across loops of different lengths indicated that loop strength increased slightly after ZBTB21 depletion (Figure 4D; Supplementary Figure S3B). GSEA showed that IAA-induced loops were more enriched in larger loops (Figure 4E). Accordingly, the average loop size increased from 417 kb to 455 kb after IAA treatment of ZBTB21-degron cells (Supplementary Figure S3C). We then investigated the features of the new Hi-C loops. The results showed that the new Hi-C loops were more enriched with regulatory elements (such as CTCF/cohesin peaks, enhancers, promoters or polycomb regions) than the less ZBTB21-responsive loops (Figure 4F).

We performed chromatin interaction analyses focusing on *cis-*regulatory elements (enhancer, promoter and CTCF-binding regions) using the ZBTB21 depletion Hi-C dataset. The results showed that ZBTB21 depletion increased the Hi-C interaction signals at promoters, enhancers, CTCF sites and superenhancers (Supplementary Figure S3D). The aggregated analysis of Hi-C interaction frequencies centered on ZBTB21-bound promoters compared with promoters without ZBTB21 binding showed that ZBTB21 depletion significantly increased 3D chromatin interactions at ZBTB21-bound promoters but not at unbound promoters (Figure 4G). For example, at highly transcribed gene loci (including Lrrc75a, Ncor1 and Pigl), the Hi-C interaction frequencies increased, and these regions also showed ZBTB21 binding and decreased cohesin binding after the depletion of ZBTB21 (Supplementary Figure S3E). At the Sall1 and Chd9 loci, we observed active transcription, ZBTB21 binding and decreased binding of cohesin after ZBTB21 depletion. The interactions between Sall1 and Chd9 increased after IAA treatment of ZBTB21-degron cells, which could be independently validated by 4C-seq after ZBTB21 depletion (Figure 4H). Although the effects of ZBTB21 on chromatin interactions are modest, they are statistically significant, which is reminiscent of a previous report that subtle changes in 3D chromatin structures can have a large functional impact, for example, on transcription (104). It seems that all cohesin peaks in these regions, independent of ZBTB binding, are smaller in IAA-treated samples than in untreated samples, suggesting that ZBTB21 may regulate cohesin loading or non-specifically modulate cohesin chromatin binding.

**Figure 5.**
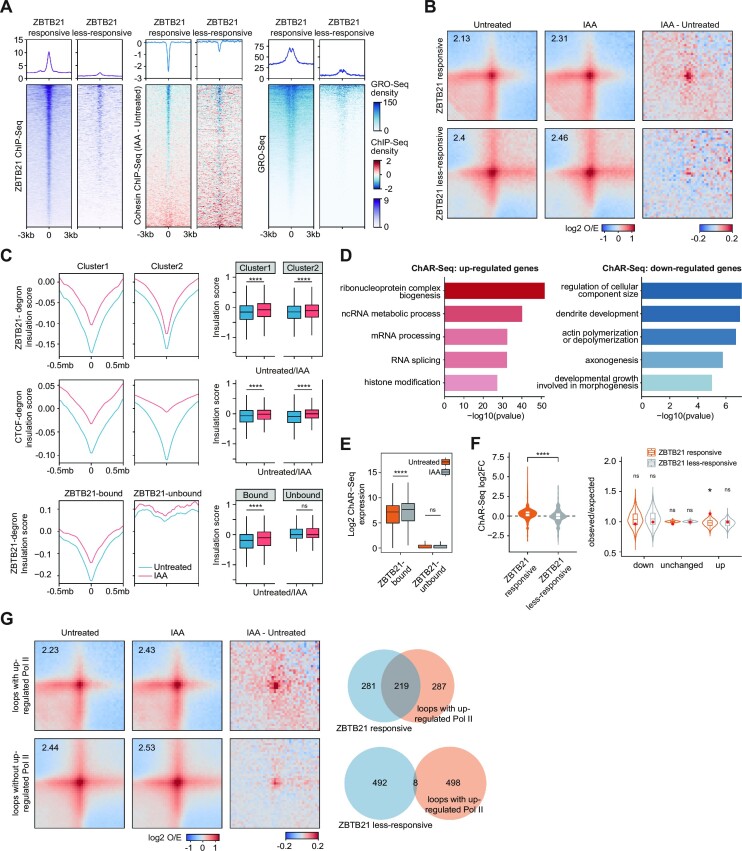
ZBTB21 depletion affects 3D chromatin interactions of ZBTB21-bound promoters. (**A**) Heatmap and aggregate signal analysis showing ZBTB21 ChIP-seq (left) and differential cohesin over 0–6 h (middle) and GRO-seq (right) centered around ATAC-seq peaks in the top 500 loops that contain both ZBTB21-bound and down-regulated cohesin peaks (ZBTB21-responsive loops) and the bottom 500 loops that do not contain ZBTB21 and down-regulated cohesin peaks (less ZBTB21-responsive loops). The color bar indicates ChIP-seq density. For the definition of responsive and less-responsive loops, see the Materials and Methods and the loop information in Supplementary Table S7. GRO-seq data were downloaded from a previous publication (128). (**B**) APA plot indicating the changes in loop strength upon ZBTB21 degradation in ZBTB21-responsive (upper panel) and less ZBTB21-responsive (bottom panel) loops. Pile-up maps were plotted at 25 kb resolution and normalized by the expected matrix. The color bar indicates the log2 ratio of the observed density/expected density. The third column shows the subtracted value of loop strength extracted from the observed/expected matrix in IAA-treated and untreated samples. (**C**) Aggregated insulation score changes around peaks in each cluster identified in Figure 2C upon ZBTB21 and CTCF degradation. Aggregated insulation score changes around ZBTB21-bound and ZBTB21-unbound promoters are shown at the bottom. The insulation scores of the ZBTB21 Hi-C maps were generated at 25 kb resolution. The insulation scores of the CTCF Hi-C maps were generated at 20 kb resolution. CTCF Hi-C data were downloaded from previous publications (25,41). Quantification results of the insulation scores are shown on the right. Significance was determined by the Wilcoxon test (****P < 0.0001). (**D**) GO analysis of up- and down-regulated genes identified by ChAR-seq upon ZBTB21 depletion. The DEGs were identified by DEseq2. (**E**) Boxplot showing the expression changes revealed by ChAR-seq for ZBTB21-bound and ZBTB21-unbound genes before and after ZBTB21 degradation. Significance was determined by the Wilcoxon test (****P < 0.0001, ns, *P* > 0.05). (**F**) The violin plots (left) show the expression changes of the genes in ZBTB21-responsive and less-responsive loops. Significance was determined by the Wilcoxon test (****P < 0.0001). The violin plots (right) show that genes in ZBTB21-responsive loops are more enriched in up-regulated genes. The observed/expected ratio was calculated by using the percentages of down-regulated, unchanged and up-regulated genes in loops (observed) normalized by the genome-wide percentage of the genes in each category (expected). The red dots indicate the observed/expected ratios of ZBTB21-responsive and less-responsive loops. ZBTB21-responsive and less-responsive loops were subjected to random permutation 10 000 times across the genome to illustrate the distributions of the ratios (violin plot), and significance was determined by the distributions. Up- or down-regulated genes were identified by DEseq2 (FDR < 0.05). (**G**) APA plot indicating the changes in loop strength upon ZBTB21 degradation in loops with (upper panel) and loops without (bottom panel) up-regulated Pol II. Up-regulated Pol II was identified by Diffbind software at gene promoters. Genes longer than 3 kb were selected for the analysis. Pile-up maps were plotted at 25 kb resolution and normalized by the expected matrix. The color bar indicates the log2 ratio of the observed density/expected density. The third column shows the subtracted value of loop strength in IAA-treated and untreated samples. Venn diagrams showing the overlap of ZBTB21-responsive loops and loops with up-regulated Pol II (upper panel) and the overlap of less ZBTB21-responsive loops and loops with up-regulated Pol II (bottom panel).

We next defined ZBTB21-responsive and less-responsive loops based on the existence of ZBTB21 ChIP-seq peaks and down-regulated cohesin peaks (Figure 5A; Supplementary Table S7). The ZBTB21-responsive loops showed higher transcriptional activity (Figure 5A). Then, the loop interaction frequencies displayed in APA plots indicated that ZBTB21 depletion caused greater increases in the loop strength of the ZBTB21-responsive loops than in that of the less-responsive loops (Figure 5B). These results suggested that ZBTB21 binding is associated with decreased cohesin occupancy and increased loop strength after the acute depletion of ZBTB21. Genomic regions with more ZBTB21 binding but less CTCF binding exhibited a greater increase in insulation scores after ZBTB21 depletion. Regions with higher densities of CTCF binding but lower densities of ZBTB21 binding displayed a greater increase in insulation scores after CTCF depletion (Figure 5C). The insulation scores at ZBTB21-bound promoters also showed a more significant increase than those at promoters not bound by ZBTB21 after ZBTB21 depletion (Figure 5C). The insulation scores of ZBTB21-unbound promoters also appeared to be sensitive to ZBTB21 depletion, indicating that the effects of ZBTB21 on 3D chromatin interactions were not specific. The GO enrichment analysis of the DEGs showed that the up-regulated genes were enriched in terms such as ribonucleoprotein complex biogenesis, mRNA processing and histone modification, and the down-regulated genes were enriched in regulation of cellular component size, dendrite development and actin polymerization or depolymerization (Figure 5D). We then explored the correlation between gene expression and the chromatin occupancy of ZBTB21. We found that the expression of ZBTB21-bound genes tended to increase more upon ZBTB21 depletion (Figure 5E). Genes associated with ZBTB21-responsive loops were also more enriched for up-regulated genes than those associated with less-responsive loops (Figure 5F). These results collectively suggested that ZBTB21 is associated with low levels of CTCF and active transcription, which involves the genomic distribution of cohesin and changes in local chromatin interactions between promoters and their regulatory elements within the range of several hundred kilobases. These changes in cohesin distribution and chromatin architecture have been extensively associated with cohesin effects on cell type-specific programs in many different cell systems, including mESCs and human cancer cells, as reported previously (105–107). The biological relevance of these regulatory mechanisms is worthy of further investigation in various cell systems in the future.

Further analyses indicated that 3D chromatin interactions increased more at genes with increased Pol II occupancy, and the ZBTB21-responsive loops also overlapped more with the loops of genes with increased Pol II occupancy. These results indicated that the increased long-range interactions were correlated with transcriptional activation after ZBTB21 activation (Figure 5G). However, it is still unclear whether the increased long-range interactions were the cause or consequence of transcription activation after ZBTB21 depletion.

### More ZBTB transcription factors are associated with the chromatin binding of cohesin

**Figure 6.**
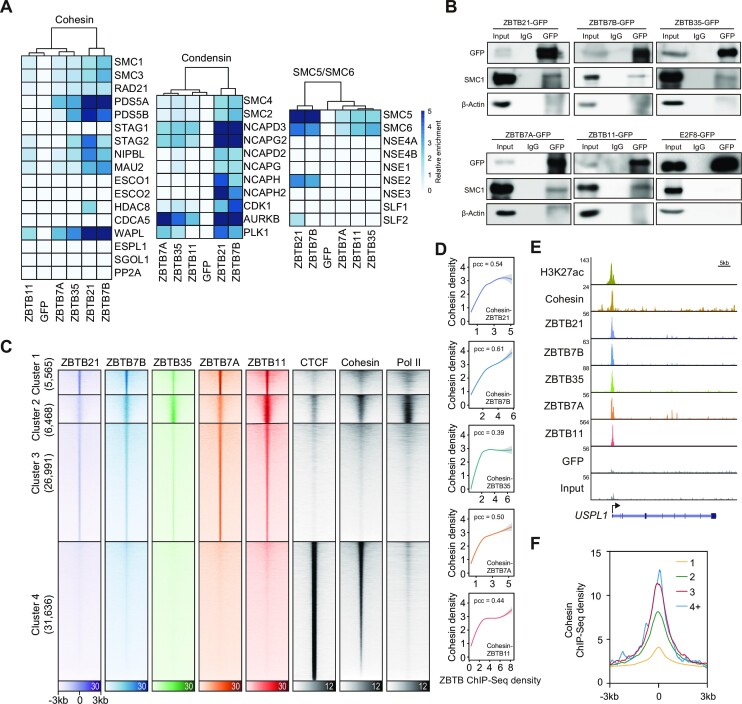
Additional ZBTB factors interact with cohesin. (**A**) Heatmap depicting the proteomic detection of the SMC complex for ZBTB protein ChIP-MS in HEK293T cells. The blue color intensity indicates the relative enrichment (IP/input). The scale bar is shown on the top right. Scale bar: relative enrichment (IP/input). The subunits of cohesin, condensin and the SMC5/SMC6 complex were based on a previous publication (129). (**B**) Western blotting was used to examine the interactions among ZBTB–GFPs and cohesin (SMC1) in native ZBTB–GFP HEK293T cells. The input cell lysate was obtained from HEK293T cells with stable Tet-on-induced expression of the protein of interest. IP samples were obtained via GFP antibody-IP. IgG and β-actin were used as negative controls. A 5% input was loaded. (**C**) Heatmap showing the occupancy of the ZBTB transcription factors cohesin, CTCF and Pol II among merged peaks of the ZBTB transcription factors cohesin and CTCF. The peaks of the ZBTB transcription factors cohesin and CTCF were first merged and then clustered by their ChIP-seq signal using the k-means method. Pol II ChIP-seq signals were plotted according to each cluster. (**D**) The curves show the correlations between ZBTB ChIP-seq density and cohesin ChIP-seq density at active gene promoters. The curves were fitted and smoothed by LOESS regression. The Pearson correlation coefficient is shown. (**E**) ChIP-seq snapshots of the co-localization among ZBTB21, ZBTB7B, ZBTB35, ZBTB7A, ZBTB11, the GFP control and the input at the USPL1 locus. (**F**) Metagene analysis indicating the cohesin ChIP-seq signals at 1/2/3/4+ ZBTB ChIP-seq peaks and cohesin peaks. 1/2/3/4+ ZBTB peaks represent the regions co-occupied by one, two, three, four or more than four ZBTB transcription factors.

There are >40 ZBTB transcription factors in mammalian cells (57,58). Inspired by the results obtained for ZBTB21, we hypothesized that ZBTB transcription factors might generally function together with cohesin. We therefore chose more essential ZBTB transcription factors based on their CRISPR scores (94) or cell type-specific functions (59,108), including ZBTB7A, ZBTB7B, ZBTB11 and ZBTB35, and generated GFP-tagged stable HEK293T cell lines with inducible expression of these four factors as well as ZBTB21 (Supplementary Figure S4A). For side-by-side comparison of the protein interacting partners among different ZBTB factors, clones with relatively similar, reasonable (not too high or too low) expression levels were used for downstream analyses (Supplementary Figure S4B). We then performed GFP ChIP-MS analysis of these cell lines. The results showed that these ZBTB transcription factor-interacting partners shared many transcription factors and chromatin regulators (Supplementary Figure S4C). Surprisingly, ZBTB ChIP-MS also identified many components of cohesin, condensin and SMC5/SMC6 complexes that were not found by GFP ChIP-MS performed in a cell line that expressed only GFP as a negative control (Figure 6A; Supplementary Table S2). IP-western blotting experiments further validated the interactions of these components with cohesin (Figure 6B). E2F8 IP did not detect SMC1 [E2F8 was shown not to connect to cohesin components in a previous study (109)]. Furthermore, we expressed these ZBTB factors with a smaller tag (HA tag) in HEK293T cells, and HA antibody immunoprecipitation followed by western blotting showed that these ZBTB factors pulled down cohesin. Cohesin IP in HEK293T cells reciprocally verified these interactions (Supplementary Figure S5A). Together, these results indicate that ZBTB transcription factors specifically interact with cohesin.

ChIP-seq experiments were then performed for ZBTB7A, ZBTB7B, ZBTB11, ZBTB21 and ZBTB35. Their genomic binding sites were distributed at promoters, enhancers and CTCF sites in the genome, and this binding could be validated by ChIP-qPCR with two different antibodies (Supplementary Figure S5B), suggesting that we captured specific binding sites of these ZBTB factors. The analyses of genomic distributions, motifs and GO functions indicated that different ZBTB factors recognized specific DNA sequences and were associated with diverse biological functions (Supplementary Figure S6A–E). Interestingly, the ZBTB21 genomic distribution and GO functions seemed to differ between 293T cells and mESCs. Furthermore, the chromatin binding strength of these ZBTB factors was positively correlated with the expression levels of the target genes, which was consistent with previously known roles of ZBTB in recruiting transcriptional regulators (Supplementary Figure S6A–E) (108,110,111).

Low levels of cohesin were observed at active promoters where ZBTB factors co-localized, but the highest cohesin binding was observed at CTCF sites where ZBTB factor binding was very weak (Figure 6C–E), and the chromatin binding strength was positively associated with the occupancy of active promoters by cohesin (Figure 6D). Our k-means clustering analyses indicated that the five ZBTB transcription factors appeared to co-localize in the genome but also revealed preferentially occupied sites (Figure 6C). ZBTB11 and ZBTB35 showed preferential binding clusters and stronger co-localization with Pol II, consistent with the recent identification of their functions in transcriptional regulation (112). We predicted that if other ZBTB transcription factors affected cohesin similarly to ZBTB21, then sites showing higher levels of ZBTB factor binding would also show more cohesin binding. Indeed, cohesin binding gradually increased as more ZBTB factors were bound (Figure 6F).

### Acute ZBTB21 and ZBTB7B depletion leads to a further decrease in cohesin occupancy

**Figure 7.**
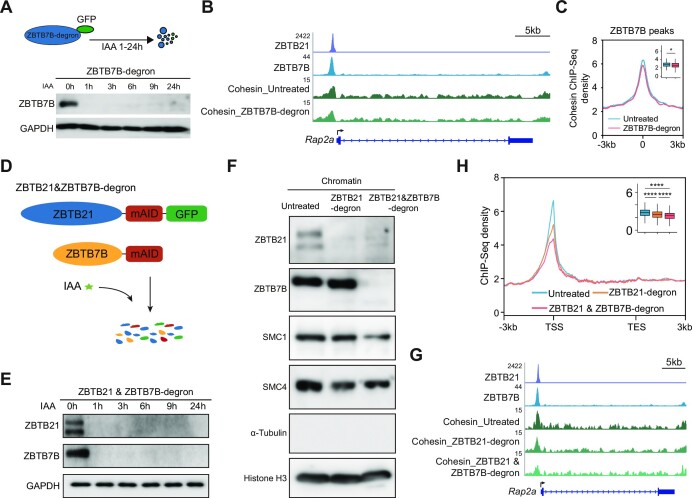
ZBTB21 and ZBTB7B are redundantly responsible for the chromatin binding of cohesin. (**A**) Western blotting analysis of ZBTB7B degradation in ZBTB7B-degron–GFP mESCs after IAA treatment at different time points. ZBTB7B was identified with a GFP antibody because it was endogenously GFP tagged, and GAPDH served as a loading control. (**B**) Snapshots of cohesin ChIP-seq signals at the Rap2a locus in untreated and ZBTB7B-depleted conditions. (**C**) The metagene displaying the cohesin ChIP-seq signals at ±3 kb around the ZBTB7B binding peaks under untreated and ZBTB7B depletion (IAA-treated) conditions. The lines indicate the mean cohesin ChIP-seq signal under each condition. The boxplot (insert) shows the changes in the ChIP-seq signal at ±100 bp around ZBTB7B peaks upon ZBTB7B degradation. Significance was determined by the Wilcoxon test (*P < 0.05). (**D**) Diagrammatic illustration of the double degradation of ZBTB21 and ZBTB7B. (**E**) Western blotting analysis of ZBTB21 and ZBTB7B protein degradation in ZBTB21 and ZBTB7B double-degron mESCs. ZBTB21 was detected with a GFP antibody because it was endogenously GFP tagged, ZBTB7B was identified with endogenous antibodies, and GAPDH served as a loading control. (**F**) Western blotting analysis of the chromatin fractions of targeted proteins in the ZBTB21-degron and ZBTB21 and ZBTB7B-degron cell lines under untreated and (6 h) IAA-treated conditions. SMC1 is a cohesin component. SMC4 is a condensin complex subunit. α-Tubulin is a cytoplasmic marker. Histone H3 is a chromatin marker. (**G**) Snapshots of cohesin ChIP-seq signals at the Rap2a locus in untreated, ZBTB21-depleted and ZBTB21- and ZBTB7B-double-depleted conditions. (**H**) Meta-gene analysis of cohesin ChIP-seq density at ZBTB21-bound genes upon ZBTB21 or ZBTB21 and ZBTB7B degradation. The lines indicate the mean cohesin ChIP-seq signal under each condition. The boxplot (inset) shows the changes in the ChIP-seq signal at ±100 bp around TSS regions upon ZBTB21 or ZBTB21 and ZBTB7B degradation. Significance was determined by the Wilcoxon test (****P < 0.0001).

We chose to degrade ZBTB21 and ZBTB7B because they showed stronger ChIP-MS signals for the cohesin and condensin subunits than the other ZBTB proteins that we investigated (Figure 6A). We first degraded ZBTB7B in mESCs (Figure 7A) and then performed cohesin ChIP-seq after ZBTB7B depletion. The illustration of meta- and single-gene examples showed that ZBTB depletion led to a slight but significant decrease in cohesin binding at ZBTB7B-binding sites (Figure 7B, C). To directly investigate the functional relationships among ZBTB transcription factors, we generated a double degradation system to simultaneously deplete ZBTB21 and ZBTB7B by knocking degron tags into the C-termini of the two genes in mESCs (Figure 7D). Western blotting confirmed the simultaneous degradation of ZBTB21 and ZBTB7B (Figure 7E).

We first performed chromatin fractionation analyses after ZBTB21 and ZBTB7B depletion. Double degradation caused changes in the protein levels of chromatin-bound SMC1 and SMC4 that were not statistically significant (Figure 7F; Supplementary Figure S7A). Consistently, the total protein levels of the cohesin and condensin components remained similar to those in the whole-cell extract (Supplementary Figure S7B), suggesting that the protein levels of the cohesin and condensin components SMC1 and SMC4, at least, did not obviously change after ZBTB21 depletion. We then performed cohesin ChIP-seq after ZBTB21 and ZBTB7B depletion. The specific gene locus and meta-gene analyses indicated that double depletion caused a greater decrease in cohesin binding than the depletion of ZBTB21 alone for the ZBTB21-bound promoters (Figure 7G, H; Supplementary Figure S7C). However, cohesin binding did not show obvious changes among genes that were not bound by ZBTB21 (Supplementary Figure S7C), which is consistent with the local changes in cohesin binding after ZBTB21 depletion.

## DISCUSSION

Cohesin plays critical roles in diseases and cell identity (113,114), and its direct regulators have been functionally linked to 3D chromatin structures (25,45,47). The proteomic profiling of cohesin-interacting partners can improve our understanding of the molecular mechanisms underlying 3D chromatin organization. Here, we report dozens of cohesin-interacting proteins associated with chromatin, and we reveal that ZBTB transcription factors interact with cohesin. The depletion of ZBTB21 leads to a decrease in the occupancy of cohesin and to increased local 3D chromatin interactions for ZBTB21-bound promoters and transcriptional activation. Moreover, the double depletion of ZBTB21 and ZBTB7B causes a further decrease in cohesin chromatin binding. Our results reveal that cohesin interacts and cooperates with ZBTB transcription factors to contribute to 3D chromatin architecture and gene expression regulation.

The well-established CTCF–cohesin complex is responsible for the formation of large-scale chromatin structures, such as TADs. However, the molecular basis of small-scale chromatin interactions (such as chromatin loops associated with active genes and sub-TAD structures) has remained unclear. Many cofactors and chromatin regulators have been implicated in the formation of biomolecular condensates to mediate local chromatin structures (20,115,116). However, recent experiments involving the chemical inhibition and rapid protein degradation of these factors did not reveal obvious effects on small-scale chromatin structures (23,24,117,118). In this study, we provide evidence indicating that ZBTB21 is both physically and functionally linked to cohesin: (i) cohesin ChIP-MS analysis identified ZBTB21 (Figure 1C, G); (ii) ZBTB21 ChIP-MS and IP-western blotting confirmed the interaction of ZBTB21 with cohesin (Figure 2A, B); (iii) ZBTB21 ChIP-seq showed the co-occupancy of cohesin with ZBTB21 at promoters (Figure 2C, D); (iv) acute ZBTB21 depletion decreased cohesin and NIPBL binding at ZBTB21-bound genes (Figure 3B, E; Supplementary Figure S2E–G); and (v) rapid depletion of ZBTB21 enhanced the 3D chromatin interactions of ZBTB21-bound active regions (Figure 5A, B). The increases in chromatin interactions in certain regions might be due to the clustering of chromatin regions modified with similar histone marks after the removal of ZBTB21/cohesin-mediated insulation, which is consistent with a model of genome compartmentalization (119,120), and the induced loops were indeed enriched in H3K4me3-, H3K27me3- and H3K27ac-modified regions (Figure 4F). Our findings were also consistent with the previous observation of strengthened interactions between superenhancers after cohesin depletion (26).

We obtained 25 kb resolution Hi-C maps in the current study, which is not high enough. This could be why we did not observe robust effects on 3D chromatin loop changes. Higher resolution chromatin interaction mapping (such as Micro-C) and elucidation of the detailed protein−protein interactions between cohesin and ZBTB21 would be valuable to clarify the molecular mechanism of ZBTB21 function in the future. A previous study showed that transcription factors are enriched at local cohesin-binding sites, and the pioneer transcription factors OCT4 and SOX2 create an open chromatin conformation for cohesin binding (90). We identified many transcription factors in cohesin ChIP-MS preparations and showed decreased cohesin binding at ZBTB21-binding sites after acute depletion of ZBTB21, which is consistent with the results described by Liu *et al.*, indicating that ZBTB21 may also function as a pioneer factor (similar to OCT4 and SOX2) to facilitate cohesin binding.

ZBTB transcription factors have been reported to function in several critical developmental processes and diseases. For example, many ZBTB transcription factors, including ZBTB7A, ZBTB7B and ZBTB35, are required for the proliferation and differentiation of T cells (59,121); ZBTB7A is involved in tumorigenesis and pluripotency control through signaling pathways or chromatin remodelers (108,122,123); ZBTB7B drives brown fat development via long non-coding RNAs (111); ZBTB11 regulates neutrophil development, and its mutation causes intellectual disability (124,125); and ZBTB21 is associated with the pathogenesis of congenital heart defects in Down syndrome (126). Their effects on 3D chromatin interactions are probably cell type specific because the distributions of ZBTB ChIP-seq peaks in mESCs and HEK293T cells are different. It would be interesting to deplete these ZBTB factors during development or disease progression in future research. The molecular mechanisms of ZBTB functions have mostly been investigated in the context of transcriptional regulation. Even though the chromatin interaction analyses were performed within 6 h, these secondary effects on mature RNAs could be limited. We could not eliminate the possibility that misregulated gene expression may also contribute to their effects on cohesin binding and 3D chromatin interactions. ZBTB21 has zinc finger domains that bind DNA, and its BTB domains mediate protein−protein interactions. Moreover, *Drosophila* insulator proteins such as Mod (mdg4) and Cp190 are also ZBTB transcription factors and have been shown to function as regulators of insulator activities (52–54). Previous studies have shown that ZBTB factors are critical for the cell fate determination of specific lineages. Although the effects of ZBTB21 depletion on cohesin chromatin binding are minor, we envisaged that ZBTB21 proteins may function as insulator proteins, similar to previously reported ZBTB factors in *Drosophila*, which might prevent cohesin extrusion at critical chromatin regulatory elements. This potential mechanism, together with transcriptional repressor activity, would be important for the specific gene expression program during different developmental processes.

## Supplementary Material

gkad401_Supplemental_Files

## Data Availability

All the data that support the findings of this study are available from the corresponding authors upon reasonable request. Raw sequencing data can be found in the GEO database: GSE184272. Access to the mass spectrometry dataset is available in ProteomeXchange: PXD028860, PXD036004, PXD035893. Raw blot, gel and microscopy image data can be found in Mendeley data: DOI: 10.17632/cwpb6kmxv7.2
