# Supplementary material for: Multiomic analysis of cohesin reveals that ZBTB transcription factors contribute to chromatin interactions
Source: Nucleic Acids Res. 2023 Jun 2;51(13):6784–805. doi: 10.1093/nar/gkad401 (PMC10359638; doi:10.1093/nar/gkad401)
Supplement: gkad401_Supplemental_Files [file gkad401_supplemental_files.zip › Supplemental Figure-20230331-.pdf]

SUPPLEMENTARY FIGURES WITH LEGENDS

Figure S1

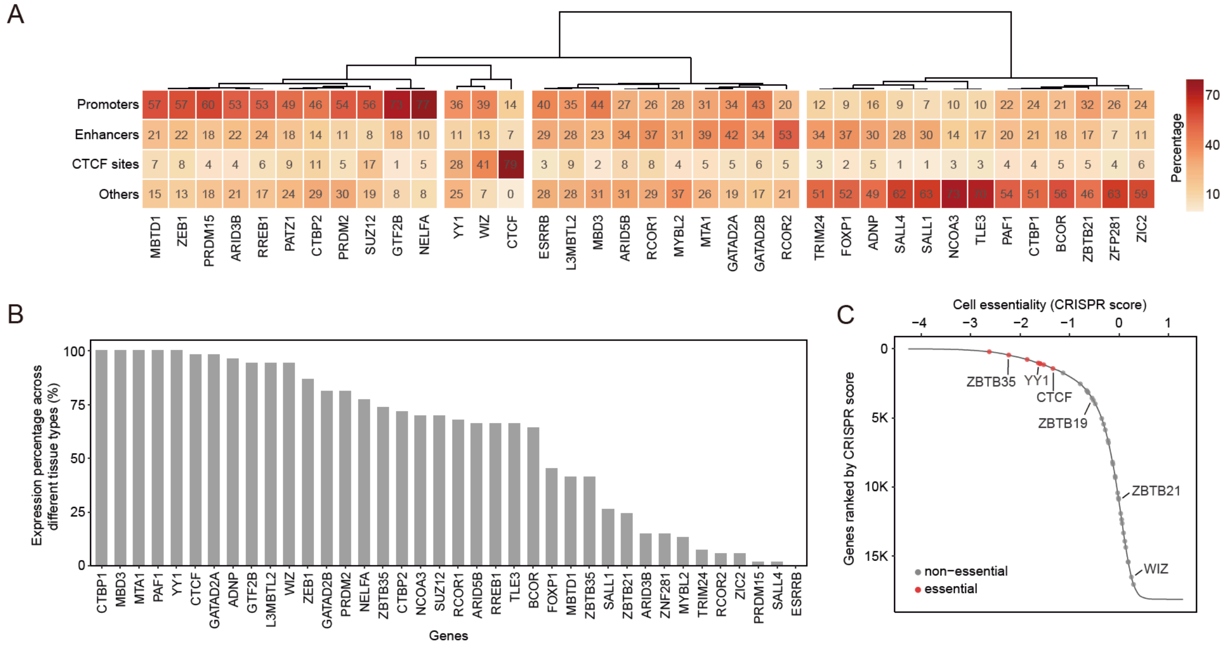

Figure S1. Characterization of proteins identified in Cohesin ChIP-MS of mESCs.

A. Heatmap showing the percentages of ChIP-seq signals of Cohesin-associated factors at cis-regulatory elements in the genome. Because many protein factors do not have reliable ChIP-seq data in mESCs, their ChIP-seq datasets were downloaded from the ENCODE or Cistrome database from different cell types (Table S8; only the factors with high-quality ChIP-seq datasets were analyzed). The promoter, enhancer, and CTCF binding regions were defined according to ChIP-seq based on H3K27ac and CTCF ChIP-seq in the corresponding cell lines. The colored bars and numbers indicate the % of binding sites.

B. Gene expression analysis of Cohesin ChIP-MS-captured transcription factors. The percent expression represents the percentages among 53 tissues that showed TPM values over 10 in the GTEx database.

C. CRISPR score analyses of Cohesin ChIP-MS-targeted transcription factors from a previous study (Wang et al., 2015). The factors in Figure S1B are indicated with dots, and essential factors are colored red. The CRISPR analyses and scores are described in the Methods section.

Figure S2

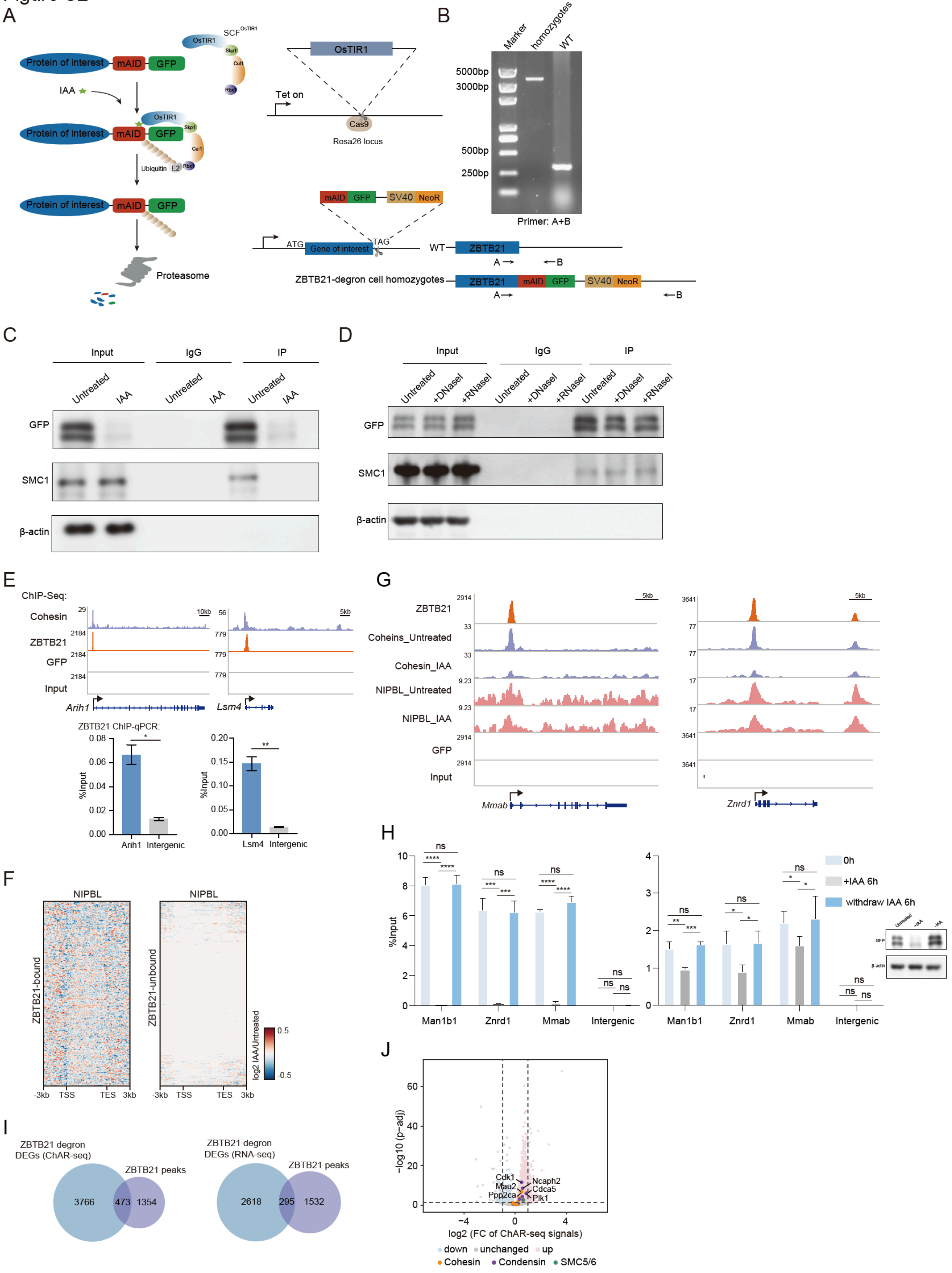
A
Protein of interest
mAID
GFP
IAA
OsTIR1
Skp1
Cul1
Rbx1
Ubiquitin
E2
Proteasome
Tet on
Cas9
Rosa26 locus
SV40
NeoR
ATG
Gene of interest
TAG
WT
ZBTB21
ZBTB21-degron cell homozygotes
B
Marker
homozygotes
WT
5000bp
3000bp
500bp
250bp
Primer: A+B
C
Input
IgG
IP
Untreated
IAA
GFP
SMC1
β-actin
D
+DNaseI
+RNaseI
E
ChIP-Seq:
Cohesin
Input
Arih1
Lsm4
ZBTB21 ChIP-qPCR:
%Input
Intergenic
F
NIPBL
ZBTB21-bound
ZBTB21-unbound
log2 IAA/Untreated
-3kb
TSS
TES
3kb
G
Coheins_Untreated
Cohesin_IAA
NIPBL_Untreated
NIPBL_IAA
Mmab
Znrd1
H
Man1b1
0h
+IAA 6h
withdraw IAA 6h
I
ZBTB21 degron DEGs (ChAR-seq)
ZBTB21 peaks
3766
473
1354
ZBTB21 degron DEGs (RNA-seq)
2618
295
1532
J
−log10 (p-adj)
log2 (FC of ChAR-seq signals)
Cdk1
Mau2
Ppp2ca
Ncaph2
Cdca5
Plk1
down
unchanged
up
Cohesin
Condensin
SMC5/6

Figure S2. Validation of the ZBTB21-degron mESCs.

A. Schematic illustration of the IAA-inducible degradation system. OsTIR1 combines with the SCF E3 ligase complex to degrade the endogenous proteins of interest. The endogenous proteins fused to the mAID tag were degraded under IAA induction. OsTIR1 was knocked into the mouse Rosa26 locus by using CRISPR/Cas9 and sgRNA. The mAID-GFP tag was knocked into both alleles before the stop codon of endogenous ZBTB21 by CRISPR/Cas9 gene editing.

B. Genotyping of selected positive degron clones. Primer designs refer to the schematic illustration below. Homozygotes are ZBTB21-GFP degron cells. WT indicates unengineered ZBTB21 cells, which served as a negative control.

C. Western blotting analyses of ZBTB21 interactions with Cohesin after ZBTB21 depletion in mESCs. IP was performed with GFP antibodies and mES cell extracts. ZBTB21-GFP without and with IAA treatment. IgG and β-actin served as negative controls.

D. Western blotting analyses of ZBTB21 interactions with Cohesin after ZBTB21 treatment by DNaseI/RNaseI with mES cell extracts. IP was performed with GFP antibodies and ZBTB21-GFP-untreated/DNaseI/RNaseI-treated mES cell extracts. IgG and β-actin served as negative controls.

E. ZBTB21 and Cohesin (SMC1) ChIP-seq tracked snapshots at the Arih1 and Lsm4 loci. GFP ChIP-seq and Input served as the negative controls. Bottom panel: ChIP–qPCR validation of ZBTB21 ChIP-seq signals in the Arih1 and Lsm4 promoter regions. The intergenic region served as a negative control. Two replicates were performed for each site. Statistical significance was evaluated by Student's t test (** $p < 0.01$, *

p < 0.05). The error bars represent SDs. The information for the qPCR primers is listed in Table S9.

F. Heatmap showing the occupancy changes of NIPBL at ZBTB21-bound and ZBTB21-unbound genes before and after ZBTB21 degradation. The color bar indicates the log2-fold changes in ChIP-seq signals between cells with and without ZBTB21 depletion.

G. ZBTB21, Cohesin (SMC1) and NIPBL ChIP-seq tracks at the Mmab and Znrd1 loci. The ZBTB21-untreated and IAA-treated conditions are shown. GFP ChIP-seq and Input served as the negative controls.

H. ChIP–qPCR validation of ZBTB21 and Cohesin (SMC1) chromatin binding to the Man1b1, Znrd1, and Mmab promoter regions under untreated/IAA-treated/IAA-withdrawal conditions. The intergenic region served as a negative control. Three replicates were performed for each site. Statistical significance was evaluated by Student's t test (**** $p < 0.0001$, *** $p < 0.001$, ** $p < 0.01$, * $p < 0.05$, ns >0.05). The error bars represent SDs. The information for the qPCR primers is listed in Table S9. Western blotting analyses of ZBTB21 expression after ZBTB21 treatment by IAA and withdrawal of IAA in mESCs are shown in the right panel.

I. Venn diagram illustrations of the ZBTB21 binding peaks with the ZBTB21 degradation-affected genes (DEGs) with ChAR-seq (left) and RNA-seq (right) data.

J. Volcano plot showing the ChAR-seq gene expression changes of Cohesin, Condensin, and SMC5/6 subunits after ZBTB21 depletion.

Figure S3

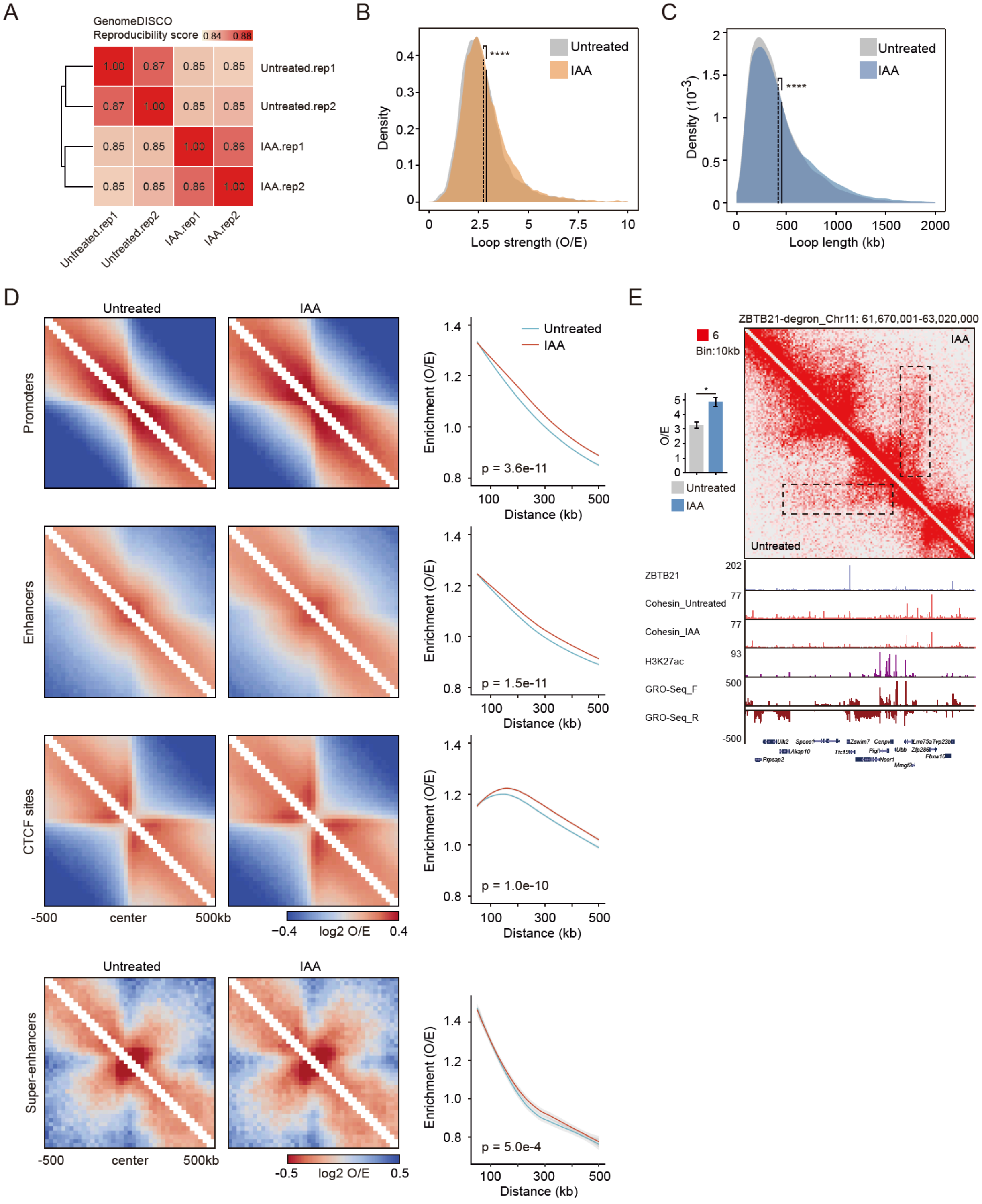

Figure S3. ZBTB21 depletion slightly increases 3D chromatin interactions.

A. Reproducibility analysis of each replicate of the samples before and after ZBTB21 degradation. The reproducibility scores were calculated by GenomeDISCO software.
B. The distribution of loop strength before and after ZBTB21 degradation. Loops are merged from the loops of untreated and IAA-treated cells. The mean loop strength under untreated (dashed line) and IAA-treated (solid line) conditions is shown in the graph.
C. The distribution of loop length before and after ZBTB21 degradation. The mean loop length under untreated (dashed line) and IAA-treated (solid line) conditions is shown in the graph.
D. Aggregate target-centered Hi-C maps showing the changes in contact strength around promoters, enhancers, CTCF sites and super-enhancers. Pile-up maps were plotted at 25-kb resolution and normalized by the expected matrix. The color bar indicates the log2 ratio of observed/expected density. The right panel shows the decay curve of the Hi-C interaction frequencies in the corresponding regions. Significance was determined by the Wilcoxon test in paired mode.
E. Hi-C contact maps for the regions of chromosome 11 (61.67–63.02 Mb) at a 10 kb resolution in untreated and IAA-treated (6 h) ZBTB21-degron cell lines, as displayed in Figure 4H.

Figure S4

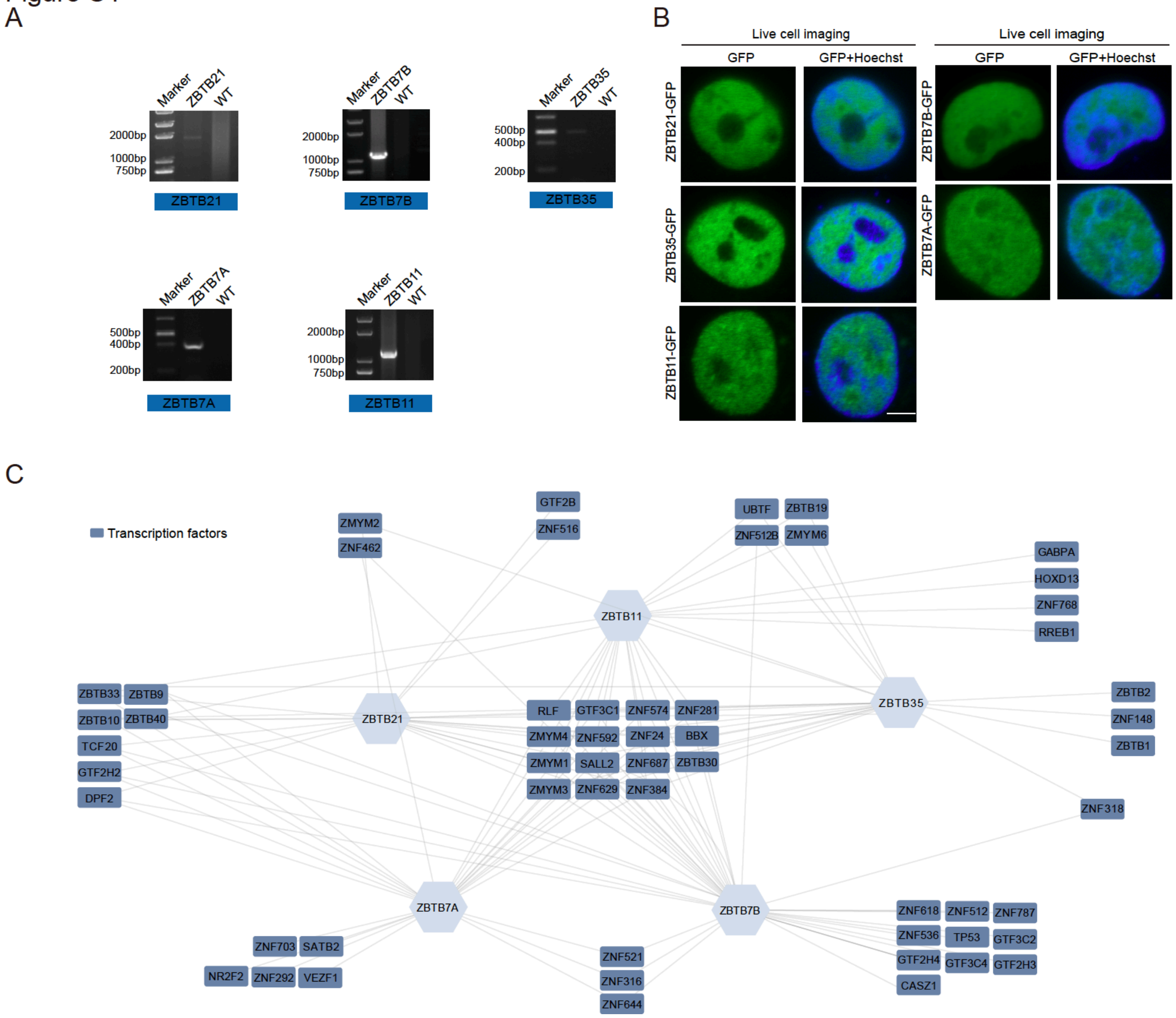

Figure S4. Validation of ZBTB-GFP stable HEK293T cell lines.

A. Genotyping verification of the knock-in of Tet-on-driven ZBTB-GFP into the AAVS1 locus in HEK293T cells. To avoid expressing very high levels of ZBTB protein, ZBTB-GFP heterozygous clones were selected for downstream analyses. WT, wild-type HEK293T cells, which served as a negative control.

B. Live cell imaging showing the ZBTB-GFP expression level. Live-cell imaging after Hoechst 33342 staining to label nuclei. Nuclear GFP fluorescence represents GFP-tagged ZBTB proteins. Scale bars, 10 μm.

C. Protein interaction network identified by ZBTB-GFP ChIP-MS in HEK293T cells. ZBTB protein bait and prey proteins (transcription factors) are indicated in the network connected by lines. The light blue nodes indicate the ZBTB proteins used for ChIP-MS. The dark blue nodes represent transcription factors identified by ZBTB ChIP-MS in this study.

Figure S5

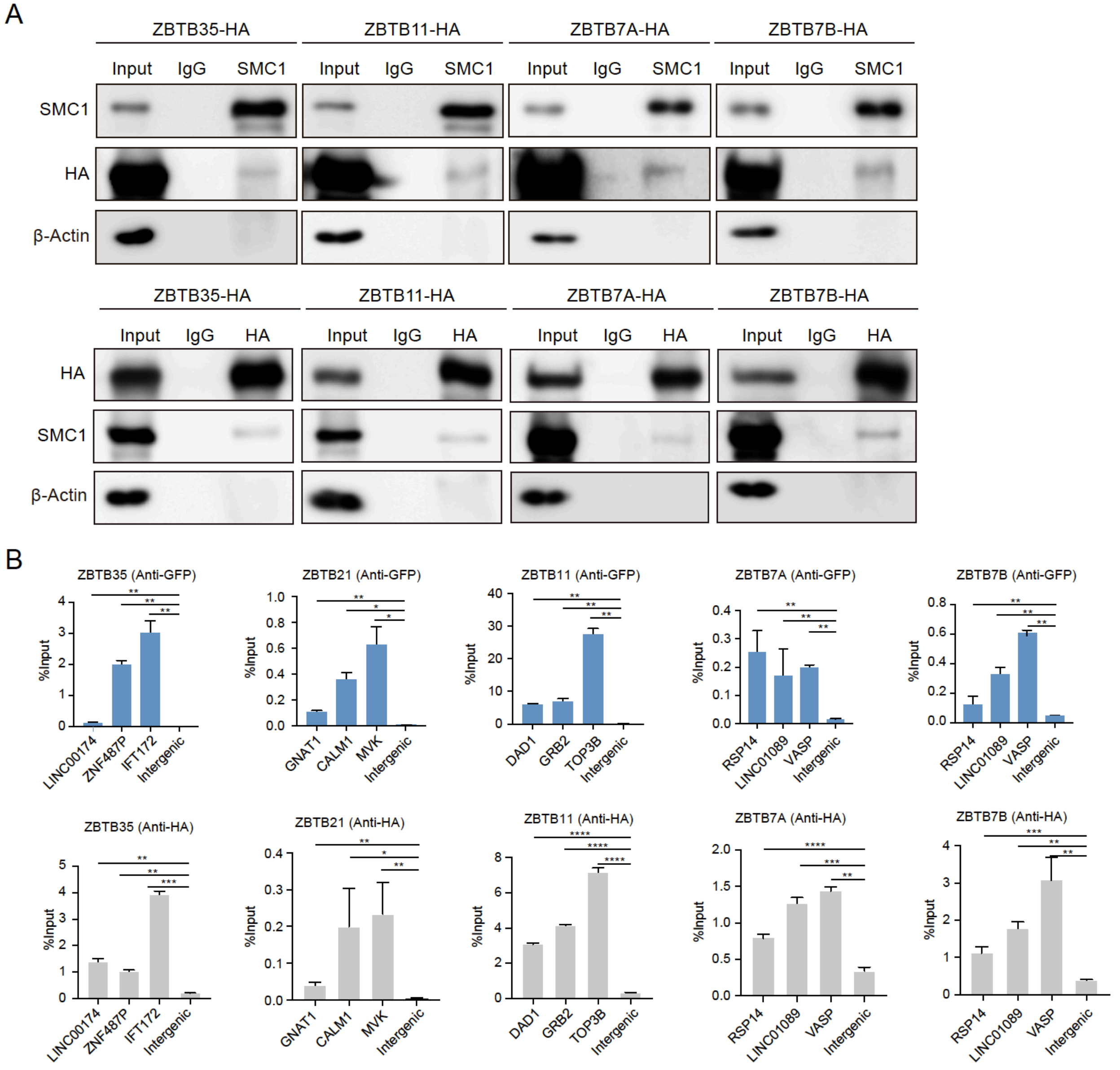

Figure S5. Validation of ZBTB factor genomic occupancy and interactions with Cohesin in HEK293T cells.

A. Western blotting was used to examine the interactions among ZBTB-GFPs and Cohesin (SMC1) in native ZBTB-GFP HEK293T cell extracts. The input cell extract was obtained from HEK293T cells with Tet-on-induced expression of the protein of interest. IP samples were obtained with an SMC1 antibody (upper panel) and HA

antibody (bottom panel). IgG and β-actin were used as negative controls. A 5% input was loaded.

B. ChIP–qPCR validation of ZBTB protein ChIP-seq signals in Figure S3B regions. ChIP experiments were carried out with GFP (upper panel) or HA antibodies (bottom panel). The intergenic region served as a negative control. Two replicates were performed for each site. Statistical significance was evaluated by Student's t test (**** $p < 0.0001$, *** $p < 0.001$, ** $p < 0.01$, * $p < 0.05$, ns: not significant). The error bars represent SDs. Information on the qPCR primers is listed in Table S9.

Figure S6

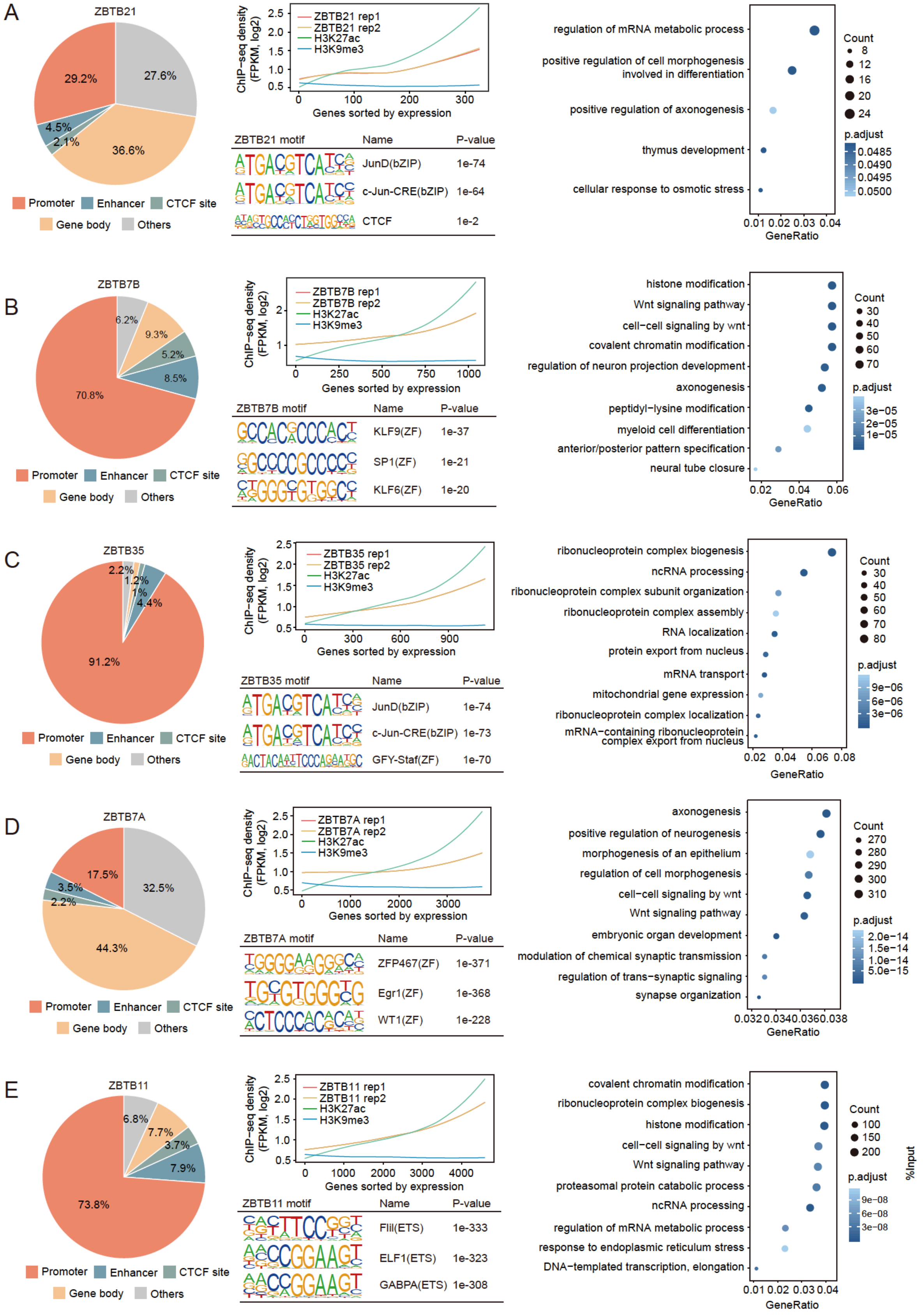

A
ZBTB21
29.2%
27.6%
4.5%
2.1%
36.6%
Promoter
Enhancer
CTCF site
Gene body
Others
ChIP−seq density (FPKM, log2)
ZBTB21 rep1
ZBTB21 rep2
H3K27ac
H3K9me3
Genes sorted by expression
ZBTB21 motif
Name
P-value
JunD(bZIP)
1e-74
c-Jun-CRE(bZIP)
1e-64
CTCF
1e-2
regulation of mRNA metabolic process
positive regulation of cell morphogenesis involved in differentiation
positive regulation of axonogenesis
thymus development
cellular response to osmotic stress
Count
p.adjust
GeneRatio
B
ZBTB7B
6.2%
9.3%
5.2%
8.5%
70.8%
ZBTB7B rep1
ZBTB7B rep2
ZBTB7B motif
KLF9(ZF)
1e-37
SP1(ZF)
1e-21
KLF6(ZF)
1e-20
histone modification
Wnt signaling pathway
cell−cell signaling by wnt
covalent chromatin modification
regulation of neuron projection development
axonogenesis
peptidyl−lysine modification
myeloid cell differentiation
anterior/posterior pattern specification
neural tube closure
C
ZBTB35
2.2%
1.2%
1%
4.4%
91.2%
ZBTB35 rep1
ZBTB35 rep2
ZBTB35 motif
JunD(bZIP)
1e-74
c-Jun-CRE(bZIP)
1e-73
GFY-Staf(ZF)
1e-70
ribonucleoprotein complex biogenesis
ncRNA processing
ribonucleoprotein complex subunit organization
ribonucleoprotein complex assembly
RNA localization
protein export from nucleus
mRNA transport
mitochondrial gene expression
ribonucleoprotein complex localization
mRNA−containing ribonucleoprotein complex export from nucleus
D
ZBTB7A
17.5%
32.5%
3.5%
2.2%
44.3%
ZBTB7A rep1
ZBTB7A rep2
ZBTB7A motif
ZFP467(ZF)
1e-371
Egr1(ZF)
1e-368
WT1(ZF)
1e-228
axonogenesis
positive regulation of neurogenesis
morphogenesis of an epithelium
regulation of cell morphogenesis
cell−cell signaling by wnt
Wnt signaling pathway
embryonic organ development
modulation of chemical synaptic transmission
regulation of trans−synaptic signaling
synapse organization
E
ZBTB11
6.8%
7.7%
3.7%
7.9%
73.8%
ZBTB11 rep1
ZBTB11 rep2
ZBTB11 motif
Fli(ETS)
1e-333
ELF1(ETS)
1e-323
GABPA(ETS)
1e-308
covalent chromatin modification
ribonucleoprotein complex biogenesis
histone modification
cell−cell signaling by wnt
Wnt signaling pathway
proteasomal protein catabolic process
ncRNA processing
regulation of mRNA metabolic process
response to endoplasmic reticulum stress
DNA−templated transcription, elongation
%Input

Figure S6. The genomic distributions, motifs and functional terms of ZBTB factor ChIP-seq in HEK293T cells.

A. Genomic distribution of ZBTB21 ChIP-seq peaks in promoters, enhancers, CTCF sites, gene bodies and other regions in HEK293T cells (left). The curve shows the correlation between active gene expression and ZBTB21 ChIP-seq density at active gene promoters. The curve was fitted and smoothed by LOESS regression (middle up). The table shows the representative enriched motifs of known chromatin structure proteins at ZBTB21 ChIP-seq peaks (middle bottom). GO enrichment terms of ZBTB21 ChIP-seq-bound genes identified by ClusterProfiler. The gene ratio represents the ratio of genes enriched for each term. The color bar indicates the adjusted p value. Circle size indicates the number of enriched genes (right).
B. Same as Fig. S4A, but for ZBTB7B.
C. Same as Fig. S4A, but for ZBTB35.
D. Same as Fig. S4A, but for ZBTB7A.
E. Same as Fig. S4A, but for ZBTB11.

Figure S7

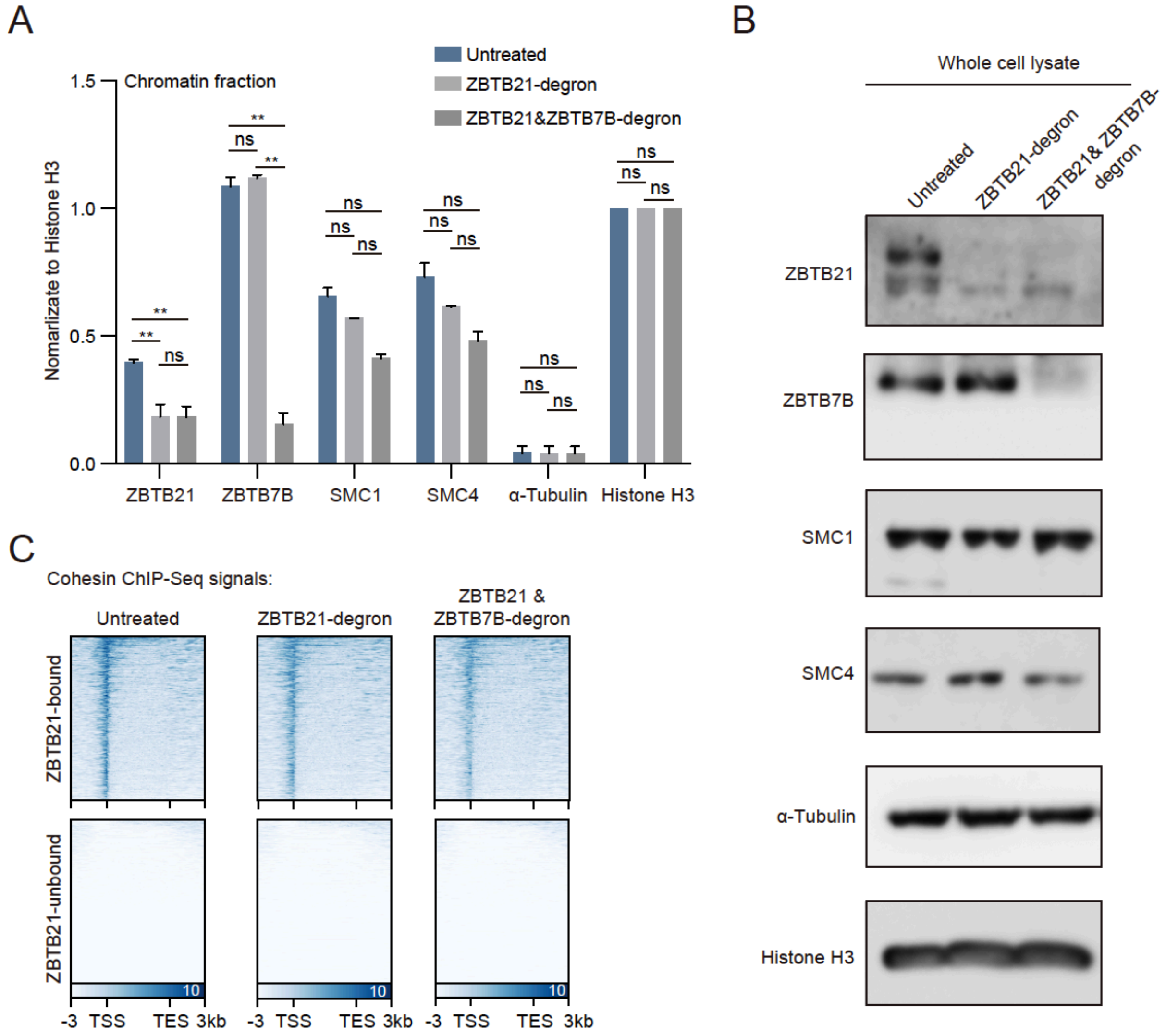

Figure S7. ZBTB21 and ZBTB7B depletion did not affect the protein level of Cohesin in mESCs.

A. The relative protein levels were quantified by using ImageJ and normalized to the histone H3 protein level. The bar graph represents the quantitative western blot grayscale normalized to histone H3. At least two replicates were calculated for each result. Statistical significance was evaluated by Student's t test (**$p<0.01$; *$p<0.05$; ns, not significant). The error bars represent SDs.

B. Western blotting analysis of the whole-cell lysis of targeted proteins in the ZBTB21-depleted and ZBTB21 and ZBTB7B double-depleted conditions (IAA for 6 h). SMC1 is a Cohesin subunit. SMC4 is a Condensin complex subunit. α-Tubulin is a cytoplasmic marker. Histone H3 is a chromatin marker.

C. Tornado plot showing the ChIP-seq signals of SMC1 at ZBTB21-bound promoters upon ZBTB21 degradation or ZBTB21 and ZBTB7B double degradation.

Supplementary Tables:

Table S1. Summary of mapping statistics of ChIP-seq, Hi-C, RNA-seq and ChAR-seq in this study.

Table S2. ChIP-MS-identified proteins in this study.

Table S3. ZBTB ChIP-seq peaks.

Table S4. Differential binding peaks of SMC1 upon ZBTB21 degradation.

Table S5. Differentially expressed genes upon ZBTB21 degradation.

Table S6. Loops identified in untreated and IAA-treated ZBTB21-degron mESCs.

Table S7. ZBTB21-responsive and nonresponsive loops.

Table S8. Publicly available datasets used in this study.

Table S9. Oligos used in this study.
